# Chemical Annotation and Biological Evaluation of Prenylated Benzophenone-Rich Fractions from *Clusia grandiflora* Fruits: Integrated In Vitro and In Silico Analyses of Antileishmanial Activity, Nitric Oxide Modulation, and Stage-Dependent Cell Death Phenotypes

**DOI:** 10.3390/molecules31162859

**Published:** 2026-08-16

**Authors:** Amanda de Jesus Alves Miranda, Caroline Martins de Jesus, Kassandra Gómez Valenzuela, José Alberto Pérez Burgos, Samuel dos Santos Soares Buna, Lívia Maria Martins Carvalho, Lucas Cardoso Marinho, José de Sousa Lima Neto, Marcelo José Dias Silva, Alberto Jorge Oliveira Lopes, Lucilene Amorim Silva, Luisa Alondra Rascón Valenzuela, Cláudia Quintino da Rocha

**Affiliations:** 1 Natural Products Chemistry Laboratory, Department of Chemistry, Federal University of Maranhão, São Luís 65080-805, MA, Brazil; miranda.amanda@discente.ufma.br (A.d.J.A.M.); samuel.buna@discente.ufma.br (S.d.S.S.B.);; 2 Parasitology and Immunology Laboratory, Center for Biological and Health Sciences, Federal University of Maranhão, São Luís 65080-805, MA, Brazil; 3 Natural Resources Research Laboratory, University of Sonora, Hermosillo 83000, Sonora, Mexicoluisa.rascon@unison.mx (L.A.R.V.); 4 Laboratory of the Angiosperm Systematics and Taxonomy Research Group, Department of Biology, Federal University of Maranhão, São Luís 65080-805, MA, Brazil; 5 Laboratory for Advanced Studies in Phytomedicines, Department of Biology, Federal University of Maranhão, São Luís 65080-805, MA, Brazil; 6 Graduate Program in Pharmaceutical Care, Department of Food and Drugs, Federal University of Alfenas, Alfenas 37130-001, MG, Brazil

**Keywords:** prenylated benzophenones, chemical annotation, antileishmanial activity, cell death phenotypes, nitric oxide modulation, molecular docking, trypanothione reductase

## Abstract

*Clusia grandiflora* Splitg. (Clusiaceae), popularly known as “cebolão” or “orelha-de-onça”, is a Neotropical species recognized as a rich source of bioactive secondary metabolites, particularly prenylated benzophenones. This study investigated the chemical composition and antileishmanial potential of the ethanolic extract (EBCG), dichloromethane fraction (FDCG), and hexane fraction (FHex) obtained from *C. grandiflora* fruits. Chemical profiling by HPLC-ESI-IT-MS/MS revealed a metabolome dominated by prenylated benzophenones, terpenes, and flavonols. Biological evaluation included activity against promastigote and axenic amastigote forms of *Leishmania* (*L*.) *amazonensis*, cytotoxicity toward RAW 264.7 macrophages, assessment of cell death phenotypes, nitric oxide (NO) production, and molecular docking against *Leishmania infantum* trypanothione reductase (TRLi). FDCG and FHex exhibited moderate activity against promastigotes (IC_50_ = 20.34 and 85.86 µg mL^−1^, respectively), whereas EBCG was inactive. FHex showed the highest activity against axenic amastigotes (IC_50_ = 7.0 µg mL^−1^), while EBCG and FDCG displayed moderate activity (IC_50_ = 15.92 and 26.43 µg mL^−1^, respectively). Cell death occurred predominantly through a necrosis-like phenotype in promastigotes and an apoptosis-like phenotype in axenic amastigotes, and all samples significantly reduced NO production. Molecular docking indicated that several putatively annotated metabolites from *C. grandiflora* can be favorably accommodated within the TS2 substrate-binding cavity of *Leishmania infantum* trypanothione reductase (TRLi). Garcinielliptone I and Guttiferone E exhibited the most favorable predicted binding affinities, whereas xanthocymol displayed an interaction pattern involving the catalytic residues Cys52 and Cys57. These findings highlight selected putatively annotated metabolites as promising candidates for future biochemical investigation. Overall, these findings highlight *C. grandiflora* fruits as a promising, previously underexplored source of prenylated benzophenones with antileishmanial activity and the ability to reduce nitric oxide production in LPS-stimulated RAW 264.7 macrophages.

## 1. Introduction

The botanical family Clusiaceae has been extensively investigated for its phytochemical and pharmacological properties, particularly in the Paleotropics [1,2]. Clusiaceae is a pantropical family comprising approximately 800 species distributed among 14 genera [3,4]. Morphologically, its members are characterized by an arboreal or shrubby growth habit, opposite leaves, and the presence of a thick white to reddish exudate [5], which plays an important ecological role in protecting the plant against herbivores and is also used by several bee species for nest construction [1]. This exudate is found throughout the plant, including the branches, leaves, and fruits, and is rich in secondary metabolites such as xanthones, terpenes, coumarins, biflavonoids, and, most notably, polyprenylated benzophenones, which are produced in the latex and floral resins and function as defensive compounds [6,7].

Among the three tribes of the family, Garcinieae has considerable commercial importance owing to the presence of edible fruits such as mangosteen and the Neotropical bacupari [8]. The tribe Symphonieae, although associated with several ethnobotanical uses [2], remains largely unexplored commercially, except in Brazil, where the edible fruit of *Platonia insignis* Mart. is widely consumed in the northern region of the country [9]. Finally, the tribe Clusieae, which includes the largest genus of the family (*Clusia* L.), has attracted considerable attention in phytochemical research [10,11,12]. Nevertheless, the large number of species highlights the need for further investigation. Owing to the remarkable structural diversity of metabolites found within the genus *Clusia*, numerous species have been reported to exhibit a wide range of biological activities, including anti-inflammatory [13], antileishmanial [14], antiviral [15], anticancer [16], anti-HIV [17], antioxidant [15] and antimicrobial activities [18].

Recent studies have investigated extracts, fractions, and isolated compounds from different species of the Clusiaceae family and demonstrated their antileishmanial activity by reducing parasite viability [19].

Leishmaniasis is a parasitic disease of major global health importance, classified by the World Health Organization as a Neglected Tropical Disease (NTD) and endemic in more than 98 countries, including Brazil [20,21]. The disease is caused by protozoan parasites of the genus *Leishmania* and is primarily transmitted through the bites of infected female phlebotomine sand flies belonging to the genera *Phlebotomus* and *Lutzomyia*. Less common routes of transmission include blood transfusion and organ transplantation. The life cycle of *Leishmania* alternates between two main developmental stages that occur in distinct hosts.

In the sand fly vector, the parasite exists as a motile extracellular promastigote, the mammalian infective stage, which is inoculated into the host skin during blood feeding together with salivary components and gut microbiota. Once in the dermis, promastigotes are phagocytosed by macrophages, within which they differentiate into intracellular amastigotes, a non-flagellated stage responsible for parasite replication and disease progression [22,23].

During *Leishmania* infection, the host mounts an immune response involving multiple inflammatory mechanisms. This response leads to the production of several pro-inflammatory mediators, among which nitric oxide (NO) plays a central role in parasite elimination. However, *Leishmania* has evolved mechanisms to manipulate macrophage signaling pathways, thereby promoting its intracellular survival within the phagolysosome. Consequently, persistent infection triggers sustained inflammation that ultimately results in the characteristic tissue damage associated with the disease. Therefore, the development of therapeutic strategies capable of not only directly eliminating the parasite but also appropriately modulating the host immune response is of considerable importance [22,24].

The clinical manifestations of leishmaniasis depend on the infecting *Leishmania* species, the host immune status, and the magnitude of the inflammatory response. Three major clinical forms are recognized: cutaneous leishmaniasis, characterized by localized ulcers that may become chronic; mucocutaneous leishmaniasis, which causes destructive inflammation of the nasal and oropharyngeal mucosa; and visceral leishmaniasis, which affects internal organs such as the liver, spleen, and bone marrow and may progress to a life-threatening systemic disease. This complexity of host–parasite interactions underscores the need for therapeutic approaches that address both parasite elimination and the host inflammatory response [25].

Conventional treatment for leishmaniasis relies primarily on pentavalent antimonials, miltefosine, and amphotericin B. However, these therapeutic options are frequently associated with severe adverse effects, high treatment costs, and the emergence of drug resistance [26]. Consequently, there is an urgent need to identify new therapeutic agents, particularly those derived from natural products, which constitute a rich source of structurally diverse secondary metabolites with considerable pharmacological potential [27].

Accordingly, given the chemical diversity and biological potential of species within the Clusiaceae family, together with the limited information available on *C. grandiflora*, an abundant species with widespread traditional use in the Amazon region, we conducted a comprehensive investigation of this species. Accordingly, this study aimed to chemically characterize extracts and fractions obtained from *C. grandiflora* fruits and to evaluate their antileishmanial activity against *L. amazonensis* promastigotes and axenic amastigotes. In addition, we assessed their cytotoxicity toward RAW 264.7 macrophages, characterized parasite cell death phenotypes, evaluated nitric oxide (NO) production, and performed molecular docking studies against *Leishmania infantum* trypanothione reductase (TRLi).

## 2. Results

### 2.1. Chemical Annotation

**Figure 1:**
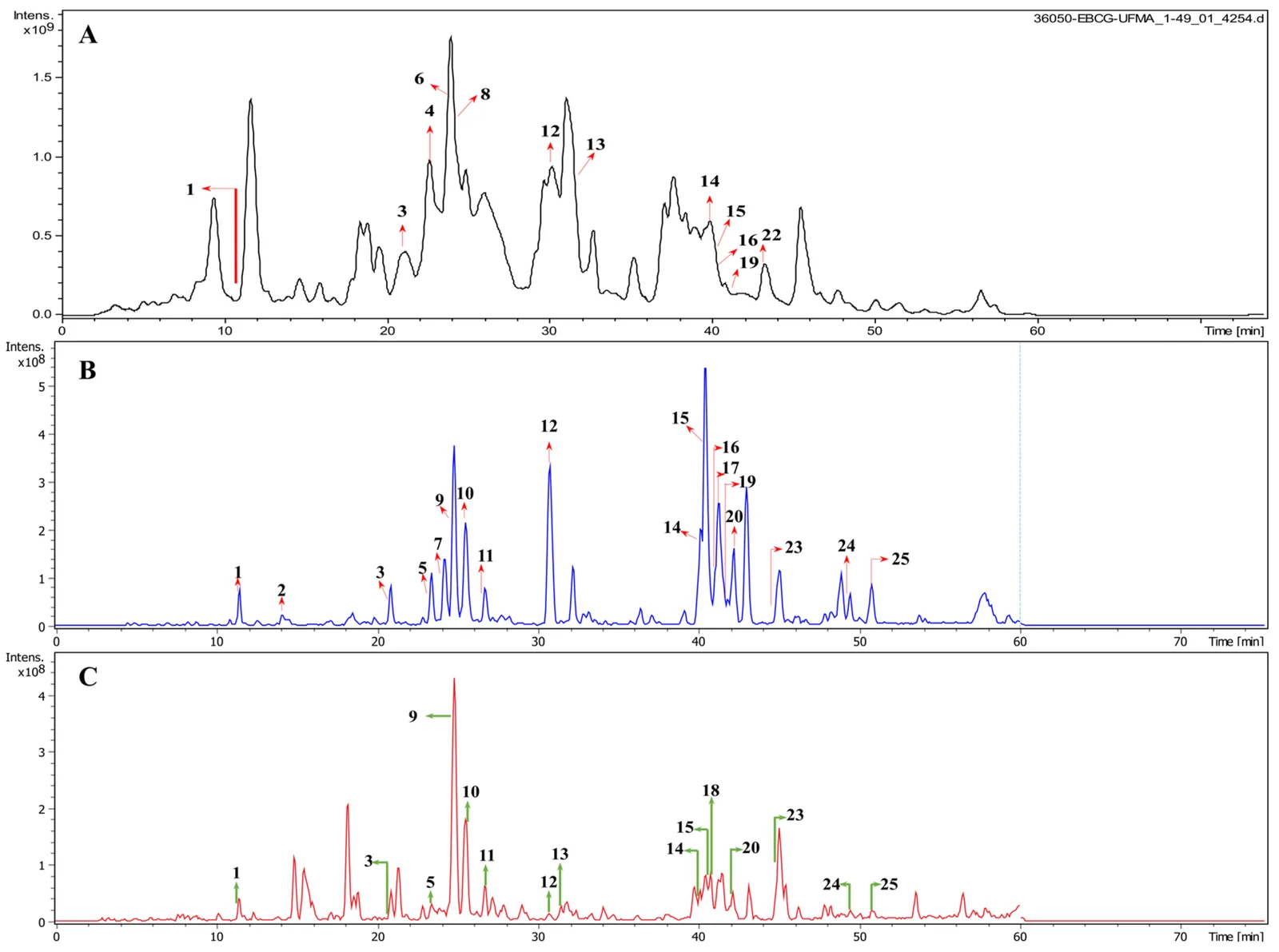
HPLC-ESI-IT-MS/MS chromatogram, positive mode, of the crude ethanolic extract (**A**) and the dichloromethane fraction (**B**) and the hexane fraction (**C**) of *C. grandiflora* fruits. Phenomenex Luna C18 column (250 × 4.6 nm; 5 µm), flow rate 1 mL min^−1^. Red and green arrows indicate the chromatographic peaks corresponding to the putatively annotated compounds, numbered according to Table S1.

**Figure 2:**
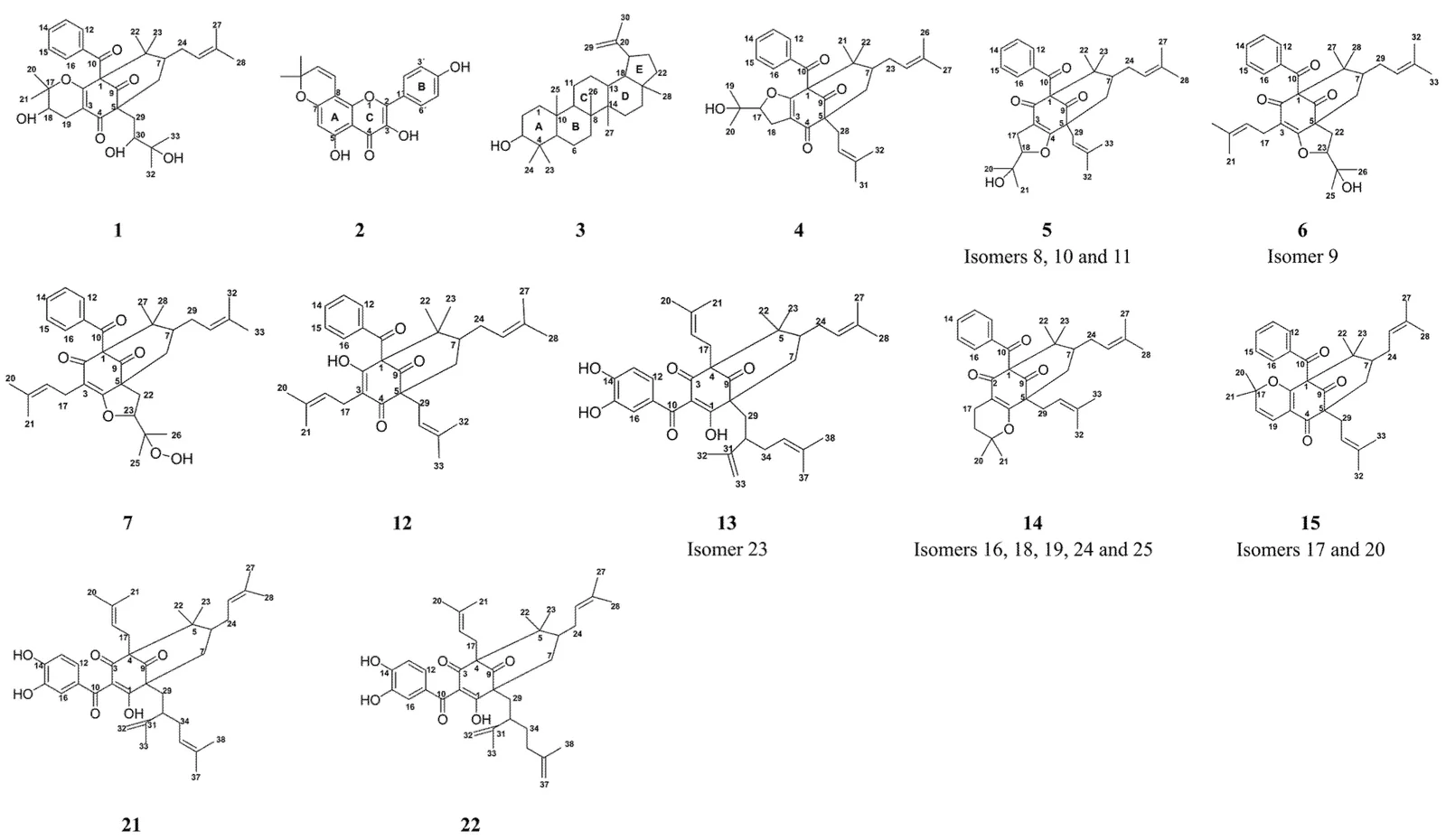
Putatively annotated compounds in the crude extract (EBCG), dichloromethane fraction (FDCG) and hexane fraction (FHex) of *C. grandiflora* fruits by HPLC-ESI-IT-MS/MS.

For the putative annotation of the detected compounds, HPLC-ESI-IT-MS/MS analyses of EBCG, FDCG, and FHex were performed in both positive and negative ionization modes. However, the positive ionization mode was selected for compound annotation because prenylated benzophenones are more readily characterized under these conditions owing to their ionization behavior (Figure 1). The proposed structures were assigned based on fragmentation patterns derived from the precursor ions obtained by multistage mass spectrometry (MS^n^) and by comparison of the HPLC-ESI-IT-MS/MS data with fragmentation profiles previously reported in the literature. This approach enabled the putative annotation of prenylated flavonols, terpenes, and predominantly polyprenylated benzophenones detected at 254 nm (Table S1). Figure 2 shows the proposed chemical structures of the putatively annotated compounds.

The compound putatively annotated as propolone B (**1**) (C_33_H_44_O_7_) was detected in EBCG, FDCG, and FHex at a retention time of 11.5 min (Figure S1 and Scheme S1, Supplementary Materials). In EBCG, the compound exhibited a protonated molecular ion [M + H]^+^ at *m*/*z* 553, whereas in FDCG and FHex it was detected as the sodium adduct [M + Na]^+^ at *m*/*z* 575. HPLC-ESI-IT-MS/MS analysis of the crude extract revealed successive losses of two water molecules, generating fragment ions at *m*/*z* 535 and 517. The fragmentation pathway subsequently followed a characteristic retro-Diels–Alder (RDA) cleavage, producing an ion at *m*/*z* 399, corresponding to the loss of a geranyl moiety (136 Da) attached at C7. Further fragmentation of the ion at *m*/*z* 399 resulted in the loss of an epoxide-derived group ([M + H − 72]^+^), yielding a fragment ion at *m*/*z* 327. Similar fragmentation profiles were observed in FDCG and FHex, indicating common fragmentation pathways among the analyzed samples. These fragmentation patterns are consistent with those previously reported for propolone B by Piccinelli et al. [28] and Hernández et al. [29], supporting the putative structural assignment.

Compound **2**, putatively annotated as prenylated kaempferol, corresponds to a kaempferol aglycone bearing a prenyl substituent [30]; it was detected exclusively in the hexane fraction (FHex) at a retention time of 14.2 min and exhibited a protonated molecular ion [M + H]^+^ at *m*/*z* 353 (C_20_H_16_O_6_). Upon MS/MS fragmentation, dehydration at the C3 position of the C ring generated a fragment ion at *m*/*z* 335 ([M + H − 18]^+^). The base peak at *m*/*z* 285 resulted from the elimination of a cyclized prenyl moiety ([M + H − 68]^+^) attached to positions C7 and C8 of ring A (Figure S2 and Scheme S2, Supplementary Materials).

Compound **3** (C_30_H_50_O), putatively annotated as lupeol, was detected in EBCG, FDCG, and FHex at a retention time of 20.8 min. The compound exhibited a protonated molecular ion at *m*/*z* 427, which underwent dehydration (18 Da) to produce the more abundant fragment ion at *m*/*z* 409. Subsequent homolytic cleavage of the C ring from the ion at *m*/*z* 409 resulted in the loss of C_16_H_26_ (218 Da), yielding the characteristic fragment ion at *m*/*z* 191 (Figure S3 and Scheme S3, Supplementary Materials) [31].

Compound **4**, putatively annotated as propolone C (C_33_H_42_O_5_), a cyclized derivative of nemorosone (12), was detected exclusively in EBCG at a retention time of 22.6 min (Figure S4 and Scheme S4, Supplementary Materials). The compound exhibited an ion at *m*/*z* 1037, consistent with a dimeric species likely formed in the ionization source. The MS/MS spectrum revealed the protonated molecular ion at *m*/*z* 519. Elimination of a prenyl group (68 Da) at C5 generated the most abundant fragment ion at *m*/*z* 451, whereas fragmentation at C7 resulted in the loss of 124 Da, producing the ion at *m*/*z* 327. This fragmentation profile is consistent with that previously reported by Piccinelli et al. [28] and Hernández et al. [29].

Compound **5**, putatively annotated as garcinielliptone I, a stereoisomer of hyperibone B (C_33_H_42_O_5_), was detected at different retention times in EBCG, FDCG, and FHex, suggesting the presence of isomeric compounds. The compound displayed fragmentation patterns similar to those observed for propolone C (Figure S5 and Scheme S5, Supplementary Materials). Fragmentation of the protonated molecular ion at *m*/*z* 519 generated the ions [M + H − 68]^+^, corresponding to the loss of a prenyl group at C5, and [M + H − 124]^+^. In all analyzed samples, the base peak was observed at *m*/*z* 451 [28,32].

Compound **6**, putatively annotated as propolone D (C_33_H_42_O_5_), was detected in EBCG at a retention time of 23.8 min and in FDCG and FHex at 24.7 min (Figure S6 and Scheme S6, Supplementary Materials). The MS/MS spectrum exhibited a protonated molecular ion [M + H]^+^ at *m*/*z* 519, followed by the loss of a water molecule, generating the fragment ion at *m*/*z* 501 ([M + H − 18]^+^). A second fragmentation pathway originated from the protonated molecular ion at *m*/*z* 519. A neutral loss of 136 Da (C_10_H_16_), likely resulting from an electronic rearrangement, produced the fragment ion at *m*/*z* 383. This fragment subsequently underwent the loss of an isobutene unit (56 Da) at the C3 position, yielding the base peak at *m*/*z* 327, which was observed in both the crude extract and the analyzed fractions.

Compound **7**, putatively annotated as propolone D hydroperoxide (C_33_H_42_O_6_), was detected exclusively in FHex at a retention time of 23.4 min (Figure S7 and Scheme S7, Supplementary Materials). In the positive ionization mode, the MS/MS spectrum exhibited a protonated molecular ion [M + H]^+^ at *m*/*z* 535. The initial fragmentation involved the neutral loss of two oxygen atoms (32 Da), generating the fragment ion at *m*/*z* 503 ([M + H − 32]^+^), which was attributed to cleavage at the C24 position. Subsequent molecular rearrangement of the ion at *m*/*z* 503, followed by fragmentation at the C3 and C5 positions, resulted in the successive elimination of two isobutene units (2 × 56 Da), yielding the fragment ion at *m*/*z* 391 ([M + H – 56 − 56]^+^). An additional fragmentation pathway was proposed from the protonated molecular ion at *m*/*z* 535, involving a neutral loss of 136 Da through a retro-Diels–Alder (RDA) cleavage, followed by the elimination of an isobutene unit from the C3 side chain to generate the fragment ion at *m*/*z* 343 ([M + H – 136 − 56]^+^). The fragmentation pattern of propolone D hydroperoxide has been previously reported by Piccinelli et al. [28].

Compound **12** was tentatively annotated as nemorosone (C_33_H_42_O_4_) and was detected in EBCG, FDCG, and FHex. MS/MS analysis revealed a protonated molecular ion [M + H]^+^ at *m*/*z* 503, which generated two main fragmentation pathways (Figure S8 and Scheme S8, Supplementary Materials). The first pathway involved the removal of the side chains at C5 and C3, resulting in the elimination of isobutene [M + H − 56]^+^ and isoprene [M + H − 68]^+^ via a mechanism involving the enol proton. These fragmentation events generated the ions at *m*/*z* 447 and *m*/*z* 435, respectively. Further fragmentation of the ion at *m*/*z* 435 led to the elimination of an additional isobutene unit [M + H − 56]^+^, yielding the ion at *m*/*z* 379. Subsequently, the loss of an isoprene unit [M + H − 68]^+^ generated the ion at *m*/*z* 311. This fragment was formed through the elimination of the side chain at C7, accompanied by the opening of the bicyclic [3.3.1] nonane system. The fragmentation mechanisms of nemorosone have been previously described by Piccinelli et al. [28] and Cuesta-Rubio et al. [33].

Three compounds exhibiting a protonated molecular ion [M + H]^+^ at *m*/*z* 603 were tentatively annotated as garcinol (**13**) (C_38_H_50_O_6_), guttiferone E (**21**) (C_38_H_50_O_6_), and xanthochymol (**22**) (C_38_H_50_O_6_) [34,35,36]. These compounds differed only in their retention times. A proposed fragmentation pathway for these compounds initially involved a neutral loss of 136 Da, resulting from ring opening between C4 and C8 through a homolytic cleavage at C4, followed by a heterolytic cleavage, generating the fragment ion at *m*/*z* 467. Subsequently, the loss of an isobutene unit ([M + H − 56]^+^) associated with C35 produced the most abundant fragment ion at *m*/*z* 411. Further elimination of an isoprene unit ([M + H − 68]^+^) linked to C4 generated the fragment ion at *m*/*z* 343. The observed fragment ions were consistent with those previously reported by Fasolo et al. [37] and Matta et al. [38].

Compound **14** was putatively assigned as propolone A (14) (C_33_H_42_O_4_). It was detected in EBCG, FDCG, and FHex, exhibiting a protonated molecular ion [M + H]^+^ at *m*/*z* 503. Based on the molecular ion, three fragmentation pathways were proposed. In the first pathway, the loss of an isobutene unit ([M + H − 56]^+^), attributed to a retro-Diels–Alder (RDA) mechanism involving the pyran ring, generated the fragment ion at *m*/*z* 447. In the second pathway, the elimination of the prenyl group attached to C5 resulted in the fragment ion at *m*/*z* 435. Subsequently, an electron rearrangement led to the neutral loss of 124 Da, generating the base peak at *m*/*z* 311 (see Figure S10 and Scheme S10, Supplementary Materials).

Compound **15** was tentatively annotated as scrobiculatone B (C_33_H_40_O_4_) and was detected in both analyzed fractions, with a retention time of 42.2 min. Analysis of the MS/MS spectrum allowed the proposal of two fragmentation pathways, both originating from the protonated molecular ion [M + H]^+^ at *m*/*z* 501 (Figure S11 and Scheme S11, Supplementary Materials). The first fragmentation pathway involved the elimination of a prenyl group [M + H − 68]^+^ at position C5, leading to the formation of the fragment ion at *m*/*z* 433. Subsequent fragmentation of the ion at *m*/*z* 433 [M + H − 124]^+^ generated the ion at *m*/*z* 309, indicating the loss of the side chain attached to position C7. Successive rearrangements, possibly involving ring opening, resulted in the formation of the fragment ion at *m*/*z* 231 [M + H − 78]^+^. A second fragmentation pathway was also proposed, in which the protonated molecular ion at *m*/*z* 501 underwent fragmentation at position C5, corresponding to the elimination of an isobutene unit [M + H − 56]^+^, yielding the ion at *m*/*z* 445. Subsequently, a neutral loss of a hydrocarbon side chain corresponding to 136 Da, attached at position C1, produced the ion at *m*/*z* 309, which corresponded to the base peak. The fragmentation pattern of scrobiculatone B has been previously reported by Trusheva et al. and Cuesta-Rubio et al. [13,39].

### 2.2. Cytotoxicity Against L. amazonensis Promastigotes

**Figure 3:**
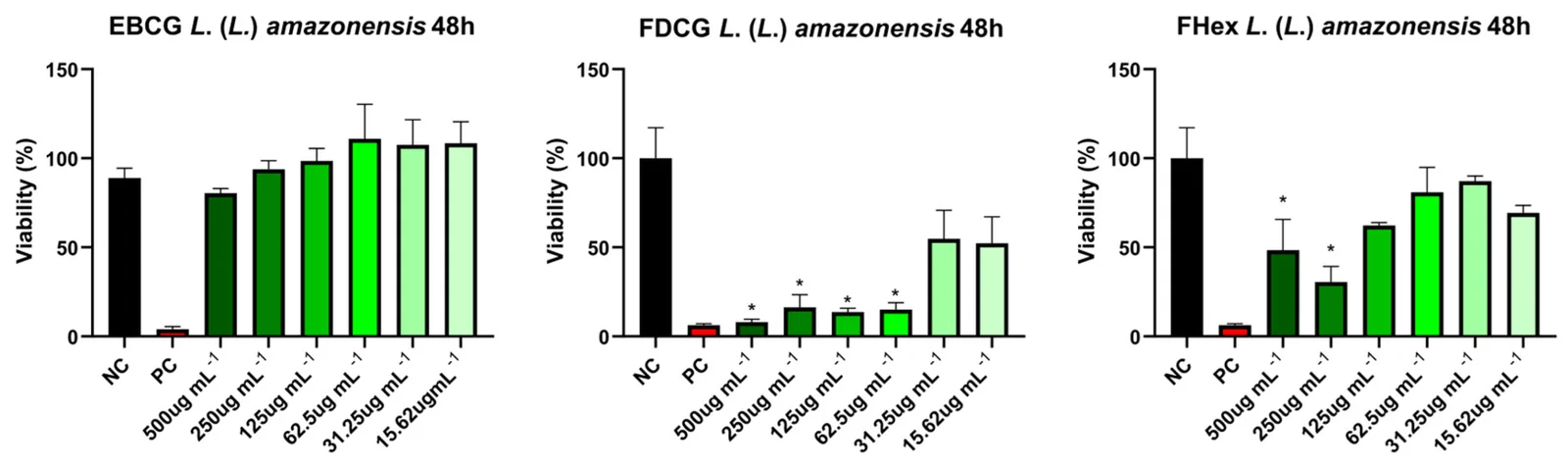
Viability of *L. amazonensis* promastigotes treated for 48 h with crude extract (EBCG), dichloromethane fraction (FDCG) and hexane fraction (FHex). NC = negative control; PC = positive control; * Asterisks indicate statistically significant differences compared to the negative control at *p* < 0.05.

FDCG demonstrated antileishmanial activity against *L. amazonensis* promastigotes, with an IC_50_ value of 20.34 µg mL^−1^, whereas FHex exhibited an IC_50_ value of 85.86 µg mL^−1^. According to the adopted classification criteria, both fractions were considered moderately active (IC_50_ = 10–100 µg mL^−1^), whereas the crude extract (EBCG) was classified as inactive (IC_50_ > 100 µg mL^−1^) [40]. Based on the IC_50_ values, FDCG exhibited approximately fourfold greater antileishmanial potency than FHex. In contrast, pentamidine, used as the positive control, exhibited an IC_50_ value of 4.0 µg mL^−1^, indicating substantially greater antileishmanial potency. Nevertheless, the activity displayed by FDCG and FHex suggests that these fractions represent promising candidates for further investigation as antileishmanial agents because of their ability to reduce parasite viability (Figure 3).

### 2.3. Cytotoxicity Against L. amazonensis Axenic Amastigotes

**Figure 4:**
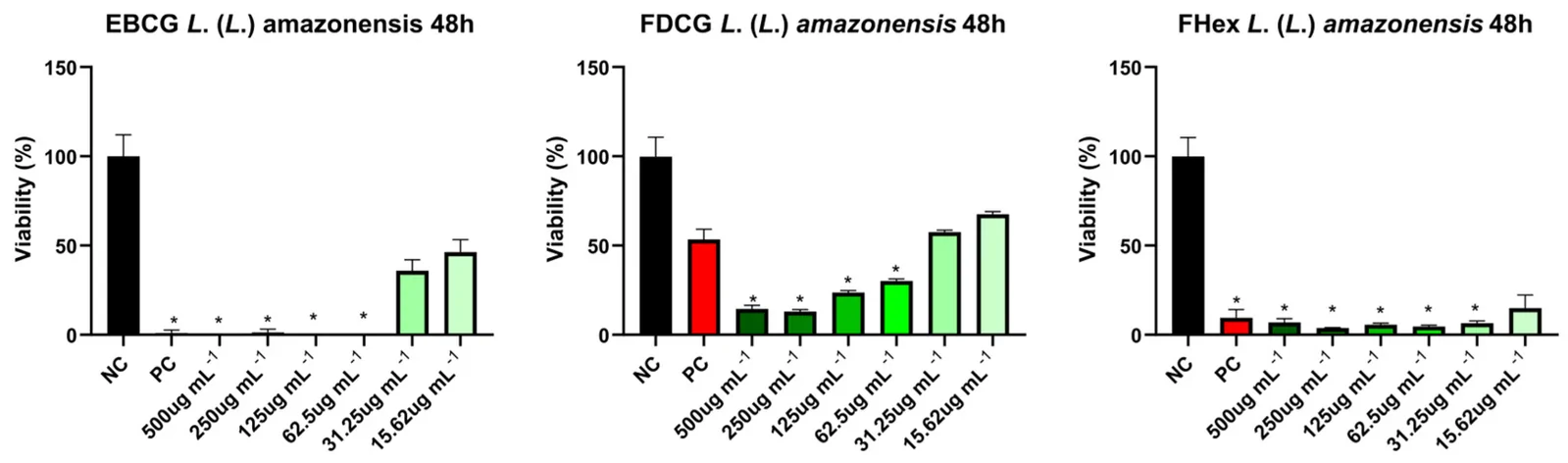
Viability of *L. amazonensis* axenic amastigotes treated for 48 h with crude extract (EBCG), dichloromethane fraction (FDCG) and hexane fraction (FHex). NC = negative control; PC = positive control; * Asterisks indicate statistically significant differences compared to the negative control at *p* < 0.05.

The crude extract (EBCG), dichloromethane fraction (FDCG), and hexane fraction (FHex) exhibited antileishmanial activity against axenic amastigotes of *L. amazonensis* (Figure 4). EBCG and FDCG exhibited IC_50_ values of 15.92 and 26.43 µg mL^−1^, respectively, and were classified as moderately active (IC_50_ = 10–100 µg mL^−1^) [40]. In contrast, FHex exhibited the highest activity, with an IC_50_ value of 7.0 µg mL^−1^, and was classified as active (IC_50_ < 10 µg mL^−1^). Among the evaluated samples, FHex was therefore the most potent fraction against axenic amastigotes [40].

### 2.4. Cytotoxicity to RAW 264.7 Macrophages

**Figure 5:**
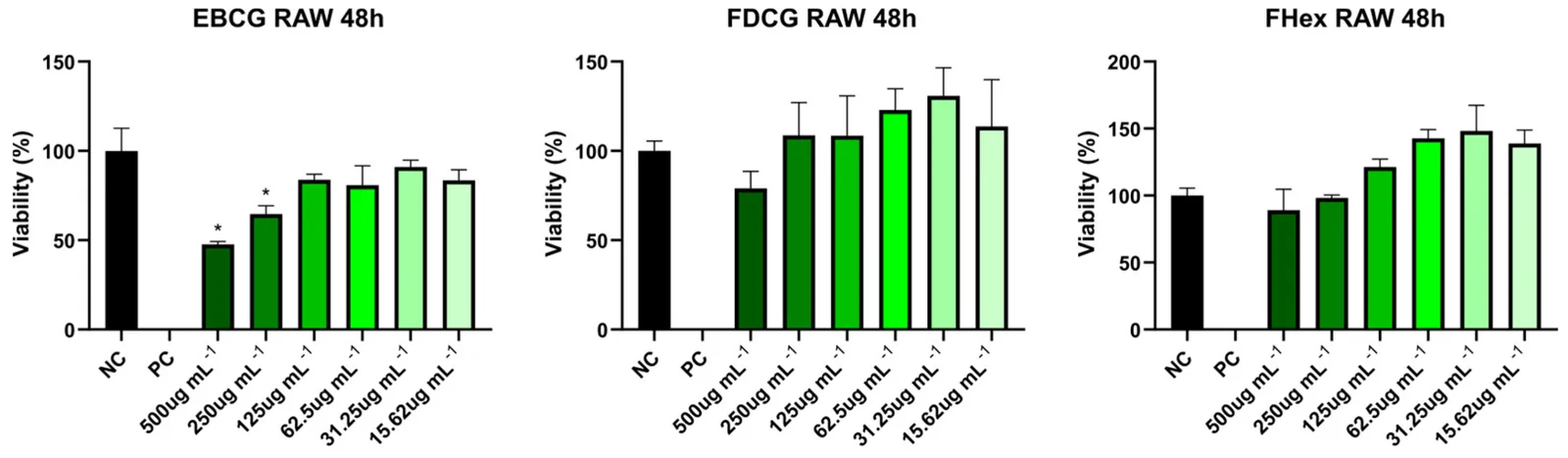
Cytotoxic activity of EBCG, FDCG and FHex was exerted against the RAW 264.7 macrophage strain treated for 48 h. NC = negative control; PC = positive control; * Asterisks indicate statistically significant differences compared to the negative control at *p* < 0.05.

The MTT assay was performed to evaluate the cytotoxicity of the crude extract (EBCG) and the dichloromethane (FDCG) and hexane (FHex) fractions toward uninfected RAW 264.7 macrophages. The CC_50_ values were 120.7, 218.0, and 134.0 µg mL^−1^ for EBCG, FDCG, and FHex, respectively (Figure 5). According to the adopted classification criteria [41], all samples were considered moderately cytotoxic (CC_50_ = 101–500 µg mL^−1^). Pentamidine, used as the positive control, exhibited a CC_50_ value of 15.24 µg mL^−1^ [42]. The concentration–response curves used to determine the IC_50_ and CC_50_ values, together with their corresponding 95% confidence intervals, are provided in the Supplementary Materials (Figures S14–S16 and Tables S2 and S3).

The selectivity index (SI) was calculated based on the CC_50_ values obtained for RAW 264.7 macrophages and the IC_50_ values determined against *L. amazonensis* (Table 1). Against promastigote forms, FDCG exhibited the highest selectivity (SI = 10.75), followed by FHex (SI = 1.56), whereas EBCG showed an SI < 0.24. Against axenic amastigotes, all samples exhibited SI values greater than 1.0. FHex showed the highest selectivity (SI = 19.2), followed by FDCG (SI = 8.27) and EBCG (SI = 7.58).

### 2.5. Cell Death

Based on the previously determined IC_50_ values for EBCG, FDCG, and FHex, parasite cell death induced by the treatments was investigated by fluorescence microscopy to evaluate apoptosis-like and necrosis-like cell death phenotypes.

**Figure 6:**
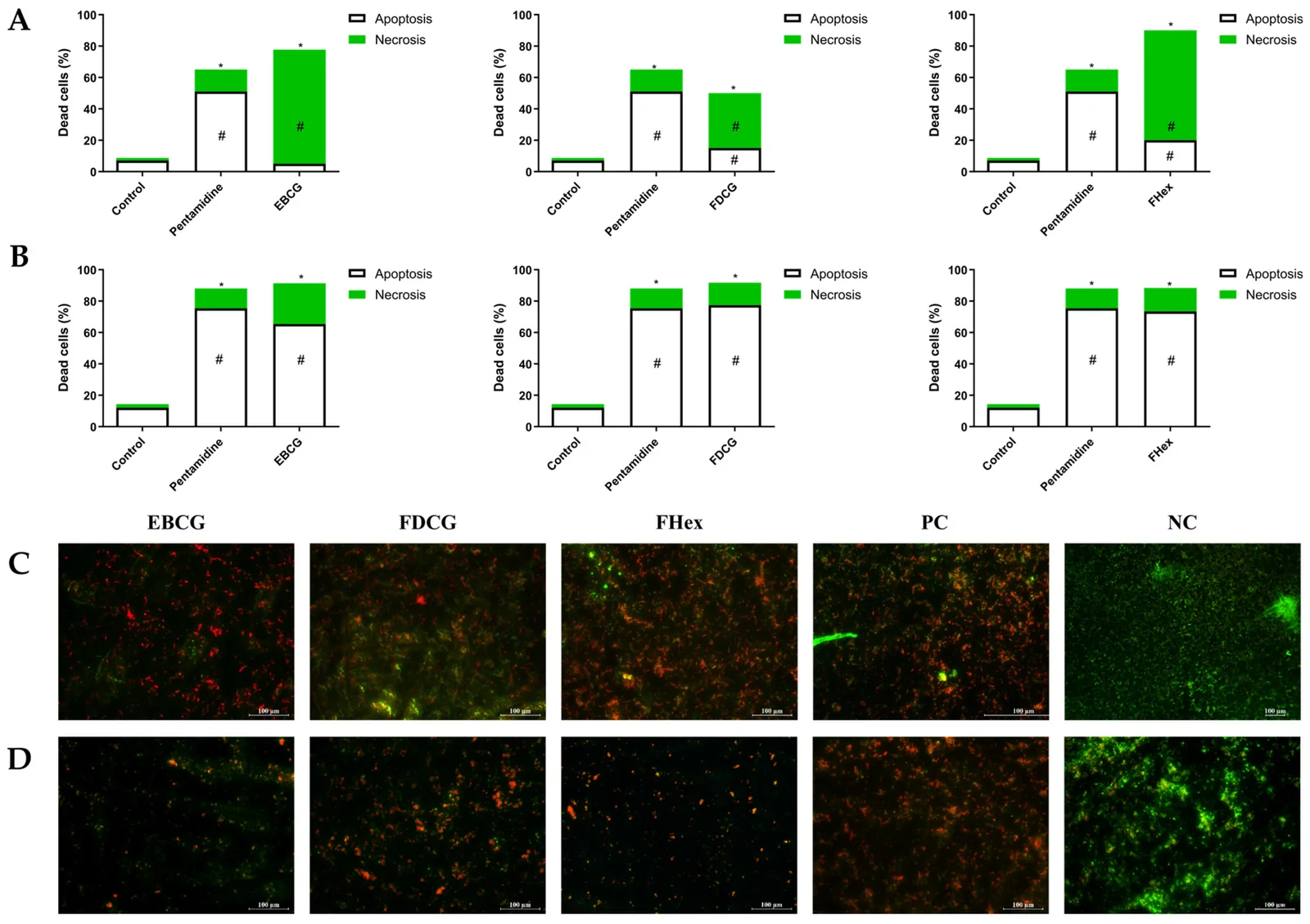
Effects of EBCG, FDCG, and FHex on the induction of cell death in *L. amazonensis.* (**A**) Percentage of cells exhibiting apoptosis-like and necrosis-like phenotypes in promastigote forms after treatment with positive and negative controls; (**B**) in axenic amastigote forms. (**C**) Representative fluorescence microscopy images of promastigote forms and (**D**) axenic amastigote forms. Showing intense red fluorescence associated with a necrosis-like phenotype and orange fluorescence associated with an apoptosis-like phenotype. Asterisks (*) indicate statistically significant differences compared with the negative control, while hash symbols (#) indicate statistically significant differences between apoptosis-like and necrosis-like cell death phenotypes.

As shown in Figure 6, EBCG, FDCG, and FHex predominantly induced a necrosis-like cell death phenotype in promastigote forms, whereas an apoptosis-like cell death phenotype predominated in axenic amastigote forms. These observations indicate differences in staining patterns between the two parasite stages but do not establish the underlying molecular mechanisms of cell death.

### 2.6. Inhibition of Nitric Oxide Production of EBCG, FDCG and FHex

The effect of EBCG and the FDCG and FHex fractions was evaluated by measuring nitric oxide (NO) production in LPS-stimulated RAW 264.7 macrophages using non-cytotoxic concentrations (cell viability ≥ 90%). As shown in Table 2, the crude extract and both fractions significantly inhibited NO production in LPS-stimulated murine macrophages. Although the IC_50_ values were similar (EBCG: 2.96 ± 0.90 µg mL^−1^; FDCG: 4.12 ± 0.67 µg mL^−1^; FHex: 2.10 ± 0.53 µg mL^−1^), statistically significant differences were observed among the treatments. Notably, FHex showed no statistically significant difference compared with the positive control, dexamethasone (IC_50_ = 1.86 ± 0.54 µg mL^−1^).

### 2.7. In Silico

**Figure 7:**
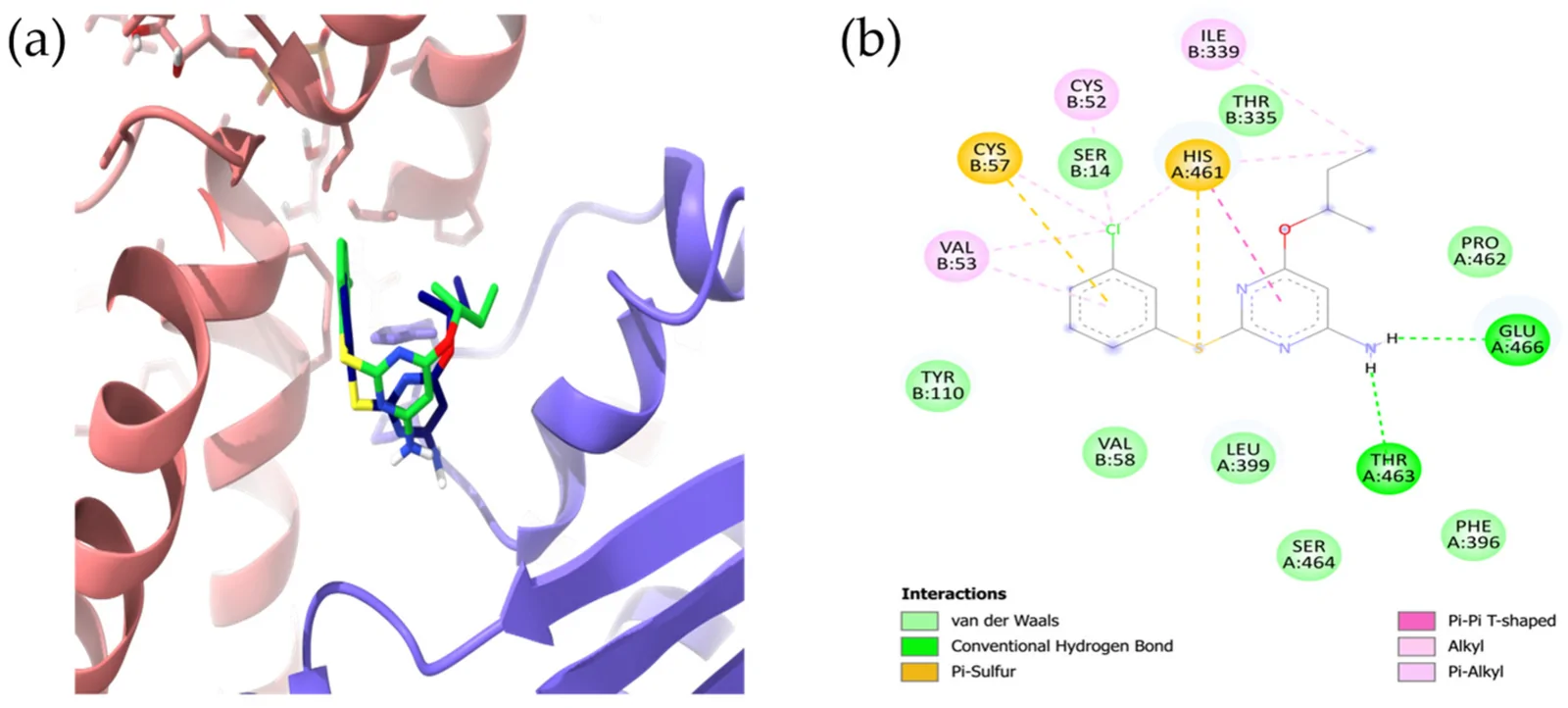
Validation of the docking protocol by redocking of RDS-777 into the TS_2_ binding site of *Leishmania infantum* trypanothione reductase (PDB ID: 5EBK). (**a**) Structural superposition of the crystallographic ligand (green) and the redocked pose (blue) within the TS_2_ pocket. (**b**) Two-dimensional protein–ligand interaction diagram of the redocked complex, highlighting the main interactions established with residues Cys52, Cys57, Val53, Ile339, His461′, and Glu466′. Colored discs represent the interacting amino acid residues, whereas dashed lines indicate the interactions between the residues and the corresponding ligand substructures.

Prior to the virtual screening analysis, the docking protocol was validated through redocking of the co-crystallized inhibitor RDS-777 into the TS_2_ binding site of *L. infantum* trypanothione reductase (TRLi). The structural superposition between the crystallographic and predicted poses yielded an RMSD value of 1.10 Å (Figure 7), indicating that the docking procedure reproduced the experimentally observed binding mode.

The docking results obtained for the compounds annotated from *C. grandiflora* are summarized in Table S4. Predicted binding affinities ranged from −6.4 to −8.0 kcal/mol. Garcinielliptone I and guttiferone E exhibited the most favorable docking scores (−8.0 kcal/mol), followed by scrobiculatone B, propolone D hydroperoxide, xanthocymol, and propolone C. In contrast, propolone B presented the lowest predicted affinity (−6.4 kcal/mol).

**Figure 8:**
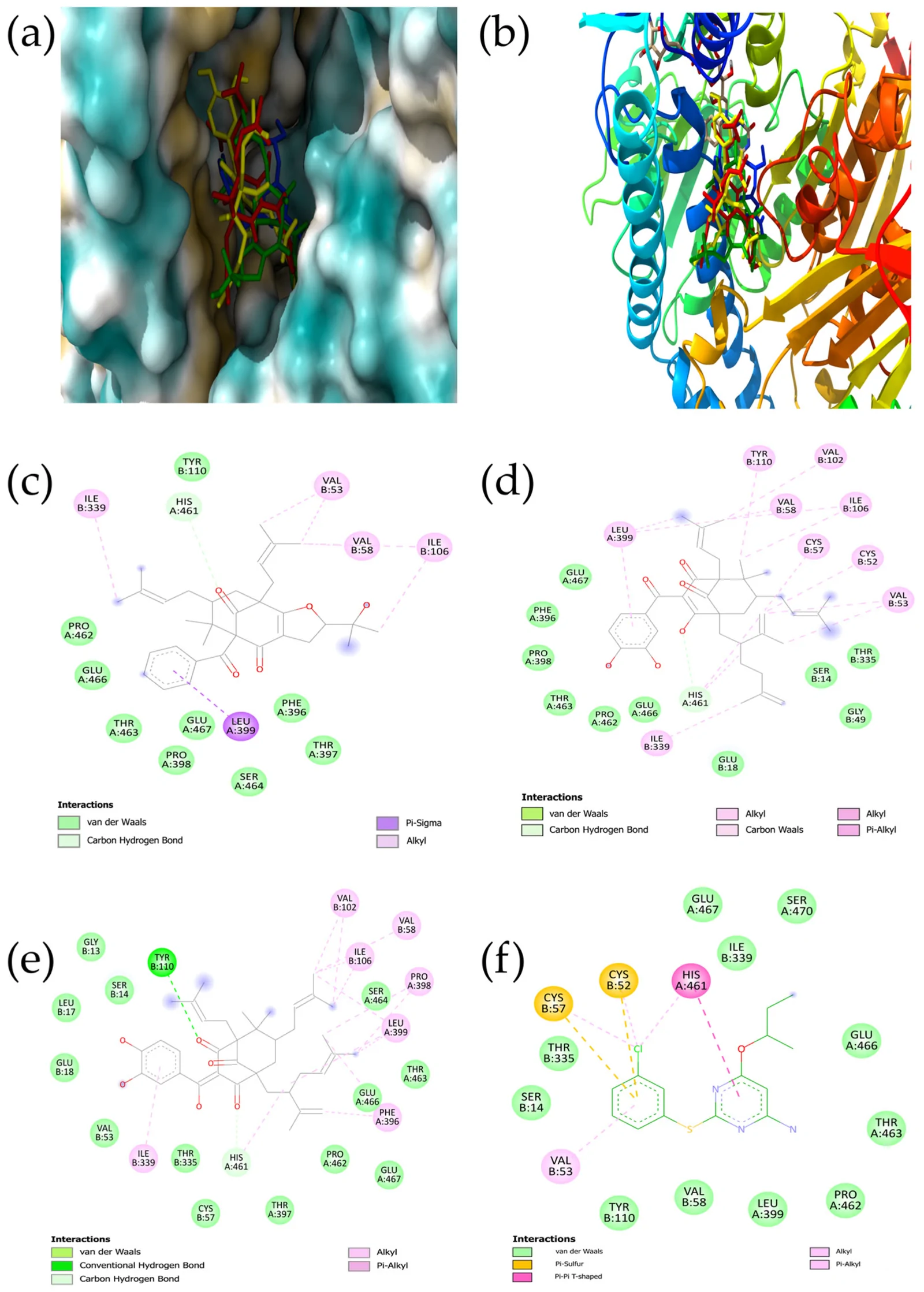
Comparative binding modes of selected metabolites and the crystallographic inhibitor RDS-777 within the TS_2_ substrate-binding cavity of Leishmania infantum trypanothione reductase (PDB ID: 5EBK). (**a**) Surface representation and (**b**) ribbon representation showing the superposition of garcinielliptone I (green), guttiferone E (yellow), xanthocymol (red), and RDS-777 (blue) within the TS_2_ cavity. Two-dimensional protein–ligand interaction diagrams of (**c**) garcinielliptone I, (**d**) xanthocymol, (**e**) guttiferone E, and (**f**) RDS-777 co-crystallized highlight the key interactions established with residues from the catalytic region and substrate-recognition regions of the TS_2_ binding site. Colored discs indicate the interacting amino acid residues, whereas dashed lines represent the interactions between each residue and the corresponding ligand substructure.

Analysis of the binding poses revealed that the compounds annotated were mainly associated with interactions established throughout the TS_2_ substrate-binding cavity (Figure 8a,b). Most compounds predominantly occupied the hydrophobic region of the binding site formed by residues Ile106, Val53, Val58, Ile339, Phe396’, Pro398’, Leu399’, and Pro462’. Interactions with these residues were consistently observed among the highest-ranked docking poses, suggesting that hydrophobic interactions contribute substantially to ligand stabilization within the cavity.

The catalytic region of TRLi, defined by the redox-active residues Cys52 and Cys57, was contacted by a more limited number of ligands. The reference inhibitor RDS-777 established interactions with both catalytic cysteines. Similar contacts were also observed for xanthocymol, whereas the remaining compounds mainly interacted with residues located in adjacent regions of the cavity (Figure 8d,f).

Several metabolites also established interactions with residues located in the substrate-recognition region of the TS_2_ cavity, particularly His461′, Thr463′, and Glu466′. The crystallographic inhibitor RDS-777 formed hydrogen-bond interactions with Thr463′ and Glu466′, whereas garcinielliptone I, propolone D, propolone D hydroperoxide, propolone A, and garcinol showed interactions involving His461′. These contacts partially reproduce the interaction pattern observed for the reference inhibitor and may contribute to ligand stabilization within the substrate-binding cavity (Figure 8c,f).

## 3. Discussion

HPLC-ESI-IT-MS/MS analysis of the hydroethanolic extract and the hexane and dichloromethane fractions led to the putative annotation of 25 compounds, most of which corresponded to structural isomers. These structural annotations were proposed based on the interpretation of HPLC-ESI-IT-MS/MS data, including molecular masses, fragmentation patterns, retention times, and comparison with previously reported literature data. Among the annotated metabolites, 23 belonged to the prenylated benzophenone class, including propolone B, propolone C, garcinielliptone I/hyperibone B, propolone D, nemorosone, garcinol, propolone A, scrobiculatone B, guttiferone E, and xanthochymol. In addition, one pentacyclic triterpene (lupeol) and one prenylated kaempferol derivative were putatively annotated. Overall, prenylated benzophenones represented the predominant class of metabolites detected in the analyzed extract and fractions.

Prenylated benzophenones exhibit characteristic MS/MS fragmentation patterns, including neutral losses corresponding to prenyl groups ([M + H − 68]^+^), isobutene ([M + H − 56]^+^), epoxide-derived moieties ([M + H − 72]^+^), and geranyl groups ([M + H − 136]^+^). These fragmentation pathways are commonly attributed to homolytic and heterolytic cleavages, as well as retro-Diels–Alder (RDA) reactions. However, structural differences among individual benzophenones may alter the observed fragmentation behavior, resulting in distinct MS/MS spectra for closely related compounds [28]. The annotation of a prenylated flavonoid further expands the chemical diversity reported for *C. grandiflora*. Flavonoids are widely distributed throughout the Clusiaceae family, particularly flavonols, which have attracted considerable attention because of their broad spectrum of biological activities [30]. Prenylation has been reported to enhance the pharmacological potential of flavonoids by increasing their lipophilicity and, consequently, facilitating membrane permeability [43].

Lupeol, a pentacyclic triterpene, was also putatively annotated in the analyzed samples. Previous phytochemical studies have reported the occurrence of terpenes in the leaves, flowers, and fruits of *Clusia* species, with triterpenes and sesquiterpenes representing the predominant classes [44]. Among these metabolites, both tetracyclic and pentacyclic triterpenes have been identified, including amyrin, friedelin, lupenone, and lanosterol, which were previously isolated from *Clusia fluminensis* Planch. & Triana [44,45].

Several species belonging to the Clusiaceae family have been reported to exhibit diverse biological activities [11]. In the present study, the dichloromethane (FDCG) and hexane (FHex) fractions exhibited moderate antileishmanial activity against *L. amazonensis* promastigotes, with IC_50_ values of 20.34 and 85.86 µg mL^−1^, respectively. Similar findings have been reported for *Platonia insignis*, a representative species of the tribe Symphonieae (Clusiaceae), which has also demonstrated promising antileishmanial activity. Compounds isolated from its hexane seed extract exhibited an IC_50_ value of 25.78 µg mL^−1^ against *L. amazonensis* promastigotes [2]. Likewise, the dichloromethane and hexane fractions obtained from the same species exhibited IC_50_ values of 2.84 and 45.23 µg mL^-1^, respectively [2,46]. Collectively, these findings reinforce the antileishmanial potential of Clusiaceae species and support the biological activity observed for the *C. grandiflora* fractions in the present study. Previous studies have suggested that the antileishmanial activity of prenylated benzophenones may be influenced by the number and position of prenyl substituents, which increase molecular lipophilicity and may contribute to both antileishmanial and cytotoxic activities [28].

The antileishmanial activity observed against axenic amastigotes is consistent with previous reports describing the antiparasitic potential of Clusiaceae species. Methanolic leaf extracts of *Symphonia globulifera* exhibited significant activity against *Leishmania donovani* amastigotes (IC_50_ = 2.1 ± 0.8 µg mL^−1^) [2], whereas the hexane extract of *Garcinia brasiliensis* exhibited an IC_50_ value of 10.66 µg mL^−1^ against *L. amazonensis* amastigotes [14]. Collectively, these findings reinforce the potential of Clusiaceae species as a valuable source of antileishmanial compounds and are consistent with the activity observed for the *C. grandiflora* samples evaluated in the present study.

Notably, the hydroethanolic extract (EBCG) and, particularly, the hexane fraction (FHex) exhibited greater activity against axenic amastigotes than against promastigotes. This difference may be related to the distinct biological and biochemical characteristics of the axenic amastigote stage. Although this experimental model does not fully reproduce the intracellular environment of the host cell, axenic amastigotes retain several stage-specific features of intracellular amastigotes, including similarities in gene expression, protein abundance, and metabolic pathways. In contrast, promastigotes possess a well-developed glycocalyx together with more efficient detoxification systems and efflux pumps, which may reduce the intracellular accumulation of xenobiotics and consequently decrease their susceptibility to antileishmanial compounds [47].

In addition to antileishmanial activity, the selectivity index (SI) is an important parameter for evaluating the therapeutic potential of candidate compounds. The SI was calculated from the CC_50_ values obtained for RAW 264.7 macrophages and the corresponding IC_50_ values against *L. amazonensis* [48]. This parameter reflects the selectivity of a sample toward the parasite relative to host cells. In general, SI values greater than 1 indicate greater toxicity toward the parasite than toward host macrophages and therefore suggest a favorable selectivity profile [41]. Furthermore, the SI provides a quantitative measure of the degree to which a compound is more selective for the parasite than for the host cell [49]. According to the SI values presented in Table 1, the dichloromethane fraction (FDCG) exhibited the most favorable selectivity profile against *L. amazonensis* promastigotes, combining high selectivity with relatively low cytotoxicity toward macrophages. The hexane fraction (FHex) also showed greater toxicity toward the parasite than toward host cells, whereas the hydroethanolic extract (EBCG) exhibited a less favorable selectivity profile because of its comparatively higher toxicity toward macrophages. These differences may be associated, at least in part, with the distinct membrane composition and cellular physiology of *Leishmania* parasites and mammalian macrophages, which can influence the uptake and intracellular accumulation of bioactive compounds [47].

Although the findings described above demonstrate the antileishmanial potential of these samples, evaluating the fluorescence staining patterns associated with parasite cell death following treatment with EBCG, FDCG, and FHex provides additional insight into the cellular responses elicited by these treatments. The predominance of a necrosis-like cell death phenotype in promastigotes and an apoptosis-like cell death phenotype in axenic amastigotes may be associated with the structural and physiological differences between these two parasite stages. Promastigotes are generally more susceptible to environmental stress and exhibit limited expression of apoptosis-associated markers, which may favor plasma membrane disruption, a characteristic feature of necrosis-like cell death [50]. In contrast, the predominance of apoptosis-like staining observed in axenic amastigotes may reflect stage-dependent differences in parasite responses to treatment. Although axenic amastigotes reproduce several characteristics of intracellular amastigotes, they do not fully mimic the intracellular host environment. Therefore, these findings should not be directly extrapolated to host–parasite interactions without additional experimental validation [51,52]. Effective treatment of leishmaniasis depends not only on parasite elimination but also on the regulation of the host inflammatory response. Although a pro-inflammatory response is essential for parasite clearance, excessive inflammation may result in tissue damage and consequently contribute to disease progression. Therefore, modulation rather than complete suppression of the inflammatory response has been proposed as a desirable therapeutic strategy in leishmaniasis [53,54]. Consistent with this concept, the *C. grandiflora* extract and fractions inhibited nitric oxide (NO) production in LPS-stimulated macrophages, with FHex exhibiting the greatest inhibitory activity. Notably, the activity of this fraction was statistically comparable to that of dexamethasone despite consisting of a complex mixture of metabolites.

Nitric oxide (NO) is widely recognized as a major effector molecule involved in the control of *Leishmania* infection, and compounds capable of promoting macrophage activation and pro-inflammatory responses are often associated with enhanced parasite clearance. However, the NO inhibition observed in the present study does not necessarily indicate that the evaluated samples favor parasite survival. During *Leishmania* infection, parasite persistence and disease progression are determined by complex interactions between the parasite and the host immune system, involving a dynamic inflammatory microenvironment composed of multiple immune cell populations rather than infected macrophages alone. Infection promotes the continuous recruitment of inflammatory cells, which contribute to maintaining this inflammatory milieu that can aggravate the disease. In this context, the present assay was designed to evaluate the effects of the samples on naïve macrophages, thereby simulating newly recruited inflammatory cells before parasite infection [24,53,55].

Previous studies have demonstrated that several plant extracts exhibiting direct antileishmanial activity are also capable of modulating inflammatory mediators in non-infected macrophages. Because NO represents only one component of the inflammatory response, its inhibition alone cannot predict the overall outcome of parasite infection. Instead, these findings suggest a potential dual therapeutic profile, combining direct antiparasitic activity with a possible modulation of the chronic inflammatory response associated with leishmaniasis [24,56]. Nevertheless, further studies evaluating additional inflammatory mediators and infected macrophage models are required to clarify the effects of these samples. At present, the results demonstrate only the potential to modulate NO production under the experimental conditions employed. The observation that NO inhibition occurred at concentrations similar to those exhibiting antileishmanial activity suggests that both effects may occur simultaneously. In agreement with this interpretation, Kolodziej et al. [57] reported that polyphenols capable of inhibiting NO production in non-infected macrophages increased NO production after macrophage activation in infected models, resulting in reduced intracellular parasite survival.

Nevertheless, it is important to emphasize that the effects of *C. grandiflora* extracts and fractions on infected macrophages have not yet been evaluated. Therefore, it cannot be excluded that the observed inhibition of NO production may either favor parasite survival or that these samples may stimulate NO production in infected macrophages, thereby contributing to parasite elimination. Consequently, studies using infected macrophages should represent the next step toward elucidating the effects of these samples within the complex immune microenvironment of *Leishmania* infection. Furthermore, because the present study was performed using murine macrophages, additional investigations employing human macrophages are required to better characterize the biological behavior of these samples and their potential relevance for human leishmaniasis [58].

Overall, the docking results showed that several metabolites putatively annotated from *C. grandiflora* can be favorably accommodated within the crystallographically validated TS2 substrate-binding cavity of TRLi. While garcinielliptone I and guttiferone E exhibited the most favorable predicted binding affinities, xanthocymol was distinguished by its interaction pattern involving the catalytic residues Cys52 and Cys57, a feature shared with the reference inhibitor RDS-777. Comparative analysis of the binding poses revealed that all selected metabolites occupied the same binding cavity, although differences in orientation and interaction profiles with catalytic and substrate-recognition residues were observed. Collectively, these interaction profiles identify a subset of metabolites with favorable structural complementarity to the TS2 cavity, supporting their prioritization for future biochemical investigation.

## 4. Materials and Methods

### 4.1. Plant Description and Distribution

**Figure 9:**
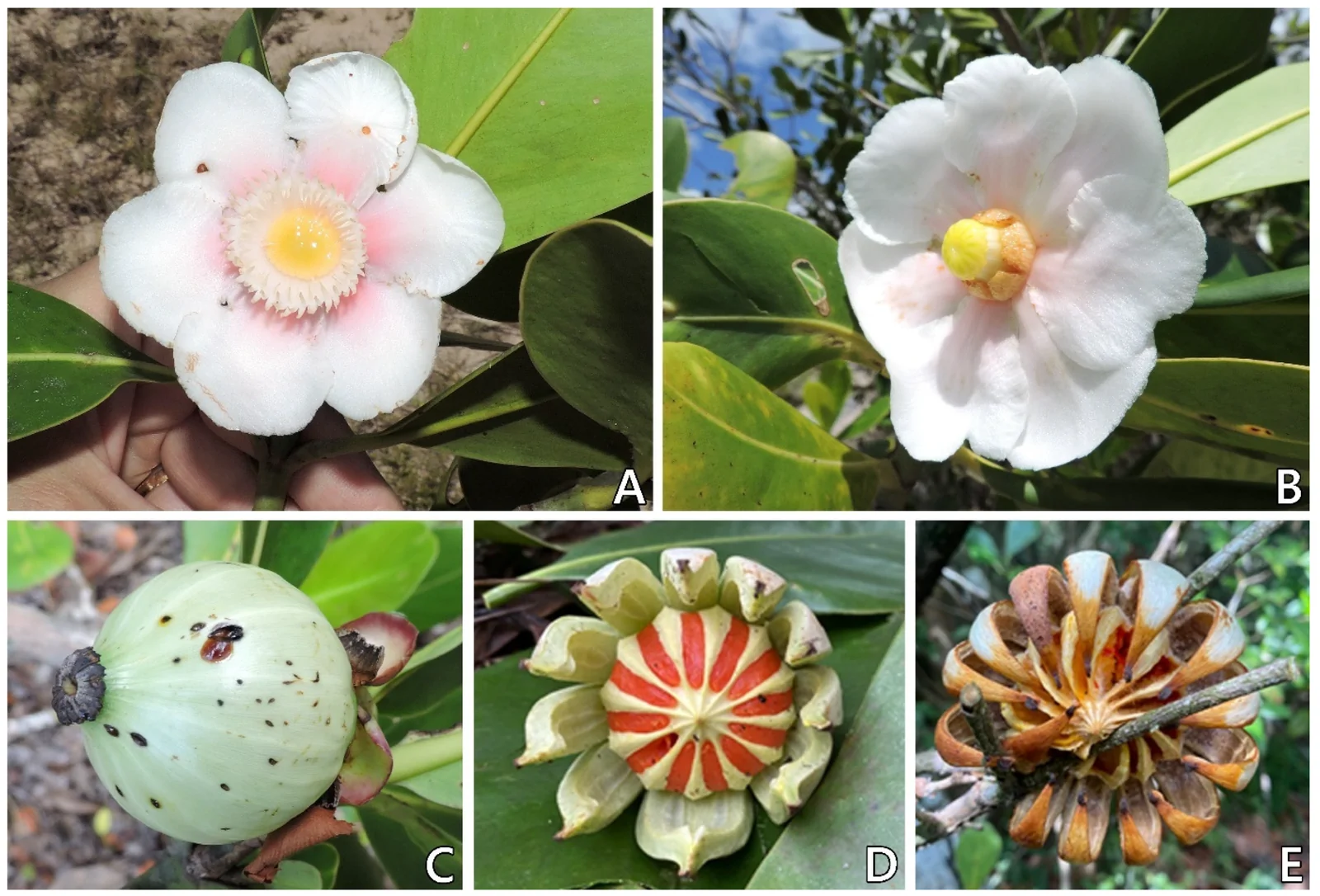
General aspect of flowers and fruits of *C. grandiflora*. (**A**) Male flower. (**B**) Female flower. (**C**) Immature fruit. (**D**) Mature fruit. (**E**) Senescent fruit. Photos: (**A**–**C**) by K.M. Pimenta; (**D**,**E**) by A.J.A. Miranda.

*C. grandiflora* is a Neotropical species distributed throughout the Amazonian Domain, typically associated with watercourses. In the state of Maranhão, *C. grandiflora* occurs in sandy areas of the northern and western regions of the state, where it exhibits a smaller growth habit than individuals found in humid forests. This is a dioecious species, bearing separate male (Figure 9A) and female (Figure 9B) flowers, with female individuals being less abundant in the population. The fruits contain an exudate, which is present in greater amounts during the immature stage (before spontaneous dehiscence; Figure 9C). In addition, the fruits of *Clusia* species are fleshy capsules that open spontaneously to expose seeds surrounded by a fleshy, sweet aril (Figure 9D). This exudate plays an important ecological role in protecting the plant against predators and is also used by several bee species in nest construction [1].

### 4.2. Material Collection and Botanical Identification

Fruits of *C. grandiflora* were collected in the Amazonian region of Maranhão State, Brazil (2°54′04.4″ S 43°55′21.3″ W). A voucher was deposited at the MG herbarium under the accession number 235737. Access to the collection of the species was registered in the Brazilian System for the Management of Genetic Heritage and Associated Traditional Knowledge (SisGen), under registration AABBC4A.

### 4.3. Extraction Procedures

#### 4.3.1. Extract Preparation and Obtaining Dichloromethane and Hexane Fractions

The fruits were dried in an oven (Hexasystems, São Paulo, Brazil) at a temperature of 40 to 50 °C and crushed in knife mills (Trapp, Santa Catarina, Brazil). The vegetable powder (1250.0 g) was subjected to extraction by exhaustive maceration with 70% ethanol (Êxodo Científica, Campinas, Brazil) at room temperature, with solvent changes every 24 h, 3 times. After extraction, the extracting liquid was filtered and subjected to rotary vacuum evaporation in a system (Vacuum pump, Model TE-058, Tecnal, Piracicaba, Brazil; Rotaevaporador, Model 802, Fisatom, São Paulo, Brazil; Ultrathermostatic bath, Model SSDu-20L, SolidSteel, Piracicaba, Brazil) at a temperature below 40 °C and subsequently lyophilized (Lyophilizer Model K105, Liotop, São Carlos, Brazil) for 48 h at a temperature of −95 °C and pressure of 12 µHg. The crude extract was solubilized in water/methanol (Êxodo Científica, Campinas, Brazil) (7:3) and partitioned six times with hexane (Êxodo Científica, FURLAB, Campinas, Brazil) and then with dichloromethane (Êxodo Científica, FURLAB, Campinas, Brazil), obtaining fractions with different polarities: the hexane fraction (2.4 g) and the dichloromethane fraction (4.85 g). After solvent removal under reduced pressure, the fractions were lyophilized under the same conditions described for the crude extract. The resulting samples were then transferred to glass vials and stored at −20 °C until further analysis.

#### 4.3.2. Chemical Profile of the Extract and Fractions by HPLC-DAD

The crude extract and fractions (5 mg mL^−1^, 1 mL) were dissolved in acetonitrile (Êxodo Científica, FURLAB, Campinas, Brazil), filtered through a 0.22 µm Millex Filter and analyzed by HPLC (Shimadzu Corp., Kyoto, Japan) with an SPD-M20A DAD detector (diode array detector). A Luna Phenomenex^®^ C18 column (C18; 250 × 4.60 mm; 5 μm; 100 Å) was used. The mobile phase A was water, while phase B was acetonitrile (HPLC grade, J.T. Baker-Avantor, Radnor, PA, USA), both acidified with formic acid (0.1%). Elution was carried out in gradient mode, from 60 to 100% B in 70 min, at a flow rate of 1.0 mL min^−1^, at 40 °C. The chromatographic separation was monitored at 254 nm.

### 4.4. Chemical Characterization by HPLC-ESI-IT-MS/MS

Chemical characterization was performed by liquid chromatography–electrospray ionization ion trap mass spectrometry using a spectrometer (amaZon SL Bruker Daltonics^®^, Billerica, MA, USA). Chromatographic analysis was performed on a 5 µm Luna C18 100 Å column (250 × 4.6 mm, Phenomenex, Torrance, CA, USA), with Prominence Shimadzu^®^ HPLC. The mobile phase was acidified with ultrapure water (0.1% HCOOH) and HPLC-grade acetonitrile—also acidified (0.1% HCOOH)—at a flow rate of 1.0 mL min ^−1^, with an acetonitrile gradient of 60–100% in 0–60 min and 100% in 60–70 min. The injection volume of each sample was 10.0 ppm.

Data acquisition was performed in positive and negative ionization modes, with multi-stage fragmentation (MS^2^ and MS^3^), according to the following parameters: nebulization gas pressure, 30.0 psi; capillary temperature, 300 °C; transfer capillary input voltage, 4500 V; desolvation gas, nitrogen (N_2_), flow rate 12 L min^−1^; collision gas, helium (He); range acquisition, *m*/*z* 50–1200. Data Analysis 4.3 software was used to acquire and process the data.

### 4.5. Culture of L. amazonensis Promastigotes

Promastigotes of *L. amazonensis* (MHOM/Br/90/BA125) were cultured in Schneider medium (Sigma-Aldrich, St. Louis, MO, USA) supplemented with 10% fetal bovine serum (FBS) (Gibco, São Paulo, Brazil), 50 µg mL^−1^ gentamicin (Gibco, Grand Island, NY, USA), 400 mg/L sodium bicarbonate (Isofar, Indaiatuba, Brazil) and 600 mg/L calcium chloride (Isofar, Indaiatuba, Brazil), at a pH of 6.8. The promastigote forms were in the stationary growth phase (4 days of culture) and exhibited flagellar motility. The culture was stored in a Biochemical Oxygen Demand (BOD) incubator (Labor, SP-500, São Paulo, Brazil) at 26 °C [59,60].

### 4.6. Differentiation of Promastigote Forms of L. amazonensis in Axenic Amastigotes

The promastigote forms (5.0 × 10^7^/mL) in the stationary phase were grown in Schneider’s medium with a pH of 5.5, supplemented with 5% FBS, 50 µg mL^−1^ gentamicin, 400 mg/L sodium bicarbonate and 600 mg/L calcium chloride to obtain axenic amastigote forms. The culture was kept at 32 °C in a BOD incubator for 96 h. The morphology of the amastigote was subsequently analyzed using Giemsa staining and microscopy.

### 4.7. Leishmanicidal Activity

Different concentrations of EBCG, FDCG, and FHex (15.62–500 µg mL^−1^) were added to *L. amazonensis* promastigotes (5 × 10^7^ cells/mL) in flat-bottom 96-well plates (KASVI, model K12-096, São José dos Pinhais, Brazil) in a final volume of 100 µL. The positive control consisted of the reference drug Pentamidine^®^ (Sanofi Aventis, Anagni, Italy), along with the parasite cultured in supplemented medium. Promastigotes cultured in Schneider medium supplemented with 10% FBSg were used as the negative control. The medium used and the diluent, 0.5% dimethyl sulfoxide (DMSO) (Isofar, Rio de Janeiro, Brazil), were also evaluated. After 48 h of incubation at 26 °C in the BOD incubator, parasite viability was assessed using the MTT assay as described in Section 4.9. The inhibitory concentration (IC_50_) was calculated based on the absorbance readings obtained.

For the axenic amastigote forms of *L. amazonensis*, the parasites (1 × 10^6^/well) were treated with serial concentrations of EBCG, FDCG, and FHex (15.62*–*500 μg mL^−1^) in triplicate and cultured for 48 h at 32 °C in supplemented Schneider medium. The negative control consisted of an axenic amastigote form culture in supplemented Schneider medium and 1% DMSO. Pentamidine^®^ (1.56–50 μg mL^−1^) was used as a positive control. At the end of the incubation period, parasite viability was assessed using the MTT assay, and the inhibitory concentration (IC_50_) was calculated based on the absorbance readings obtained.

### 4.8. Cytotoxicity Assay on Macrophages

In vitro cytotoxicity was evaluated using the RAW 264.7 murine macrophage cell line (2 × 10^6^ cells/mL), kindly provided by Dr. André Moraes Nicola (University of Brasília, Brazil), and cultured at 37 °C in a humidified atmosphere containing 5% CO_2_. The cells were exposed to different concentrations of EBCG, FDCG, and FHex (15.62–500 µg mL^−1^) in a flat-bottom 96-well plate. Untreated cells cultured in RPMI (Roswell Park Memorial Institute) medium (Aldrich, São Paulo, Brazil) supplemented with 10% FBS were used as the negative control. Cells exposed to 100% DMSO were used as the positive control, whereas cells exposed to 0.5% DMSO were used as the vehicle control. After 48 h of incubation, cell viability was evaluated using the colorimetric MTT assay. Absorbance was measured at 540 nm using a microplate reader (BioTek Instruments, Winooski, VT, USA), and CC_50_ values were calculated from the resulting cell viability data. The methodological procedures were conducted according to Tempone et al. [61] with adaptations, and the tests were performed in triplicate.

### 4.9. Determination of Cell Viability by the MTT Assay

The viability of *L. amazonensis* promastigotes, axenic amastigotes, and RAW 264.7 macrophages was evaluated using the colorimetric method MTT [3-(4,5-dimethyl-2- thiazolyl)-2,5-diphenyltetrazolium bromide] (Sigma-Aldrich, St. Louis, MI, USA). The assay was based on the reduction of the yellow, water-soluble MTT tetrazolium salt by metabolically active cells, resulting in the formation of purple, water-insoluble formazan crystals, thereby allowing cell viability to be estimated [62,63].

Following the respective treatment periods, the plates containing *L. amazonensis* promastigotes, axenic amastigotes, and RAW 264.7 macrophages were centrifuged, and the supernatant was removed and replaced with medium containing MTT (5 mg mL^−1^). The plates were then incubated for 3 h. Subsequently, the plates were centrifuged again, and the resulting formazan crystals were dissolved in 100 μL of pure DMSO. Absorbance was measured at 540 nm using a microplate reader [64]. Cell viability was determined from the absorbance values, and the resulting CC_50_ and IC_50_ values were used to calculate the Selectivity Index (S.I.), defined as the ratio of the CC_50_ value obtained for RAW 264.7 macrophages to the IC_50_ value obtained for the parasite (SI = CC_50_/IC_50_). S.I. values greater than 1 indicate greater selectivity toward the parasite than toward host cells, whereas values below 1 indicate greater cytotoxicity toward host cells.

### 4.10. Cell Death Type Assay via Acridine Orange/Ethidium Bromide (AO/EB) Labeling

To investigate the cell death phenotypes induced by EBCG, FDCG, and FHex, *L. amazonensis* promastigotes and axenic amastigotes were incubated with the IC_50_ value of each fraction for 4 h. The IC_50_ value of pentamidine and Schneider’s medium supplemented with 1% DMSO were used as positive and negative controls, respectively. After incubation, the plates were centrifuged at 3000 rpm for 10 min, and the supernatant was discarded. Then, 100 µL of acridine orange/ethidium bromide (AO/EB) dye solution (20 µg mL^−1^) was added, and the Leishmania were incubated for 10 min. Subsequently, the plates were centrifuged again, and the parasites were resuspended in 100 µL of phosphate-buffered saline (PBS). From this suspension, 20 µL were removed to prepare slides, which were analyzed under an inverted fluorescence microscope (Eclipse Ti-U, Nikon, Tokyo, Japan) at 400× magnification. A total of 100 parasites were analyzed and classified as exhibiting apoptotic-like (condensed or fragmented chromatin, green or orange fluorescence) or necrosis-like (orange chromatin turning red) phenotypes, based on EB (red fluorescence) and AO (green fluorescence) staining [65,66].

### 4.11. Statistical Analysis

Statistical analyses were performed via GraphPad Prism 7.0 software (GraphPad Software, Inc., San Diego, CA, USA). Nonlinear regression was used to determine the half-maximal inhibitory concentration (IC_50_) for *L. amazonensis* promastigotes and axenic amastigotes, as well as the cellular cytotoxicity (CC_50_) for RAW 264.7 macrophages. All experiments were performed in triplicate and repeated in three independent experiments. The concentrations were transformed into log(x) values, and the optical density (OD) was normalized to determine the relative viability (%) compared with the negative control OD. IC_50_ and CC_50_ values were determined from sigmoidal curves. The selectivity index (S.I.) was then defined as the ratio of the CC_50_ and IC_50_ values for the extract and fractions.

To assess the death pathway, the Shapiro–Wilk test was used to check for data normality. Since all variables were considered parametric, one-way analysis of variance (ANOVA) with a Tukey post hoc test was used to determine differences in the mean percentage of dead cells between groups. To compare cell death types (apoptosis or necrosis), two-way ANOVA with Tukey’s post hoc test was used. *p* values ≤ 0.05 were considered to indicate statistical significance.

### 4.12. Inhibition of Nitric Oxide in LPS-Induced RAW 264.7 Murine Macrophages

The inhibitory effect on nitric oxide production in LPS-stimulated RAW 264.7 cells was evaluated using Schmölz’s method [67]. RAW 264.7 cells (125 × 10^3^ per well, 250 μL) were seeded in 24-well plates and incubated for 24 h at 37 °C in a 5% CO_2_ atmosphere. Subsequently, the medium from each well was removed and replaced with fresh medium (125 μL), and the cells were treated with medium containing non-cytotoxic concentrations of EBCG, FDCG, and FHex (cell viability > 90%) (125 μL). After 2 h of treatment, the cells were stimulated with 1 μg mL^−1^ LPS for 24 h. Nitric oxide production was determined by measuring nitrite levels in the culture medium. An equal volume of the well supernatant (50 μL) was mixed with Griess reagent (50 μL) and incubated for 15 min in the dark. Absorbance was measured at 540 nm using a microplate reader (Thermo Fisher Scientific Inc., Miltiskan GO, Waltham, MA, USA). Nitric oxide production was expressed as a percentage, considering the vehicle-treated cells as 100%.

### 4.13. Statistical Analysis of Nitric Oxide Production

Statistical analysis for the nitric oxide assay was performed as follows: All data were expressed as the mean ± standard deviation (SD) from at least three independent experiments performed in triplicate. Statistical differences between data groups were analyzed using one-way analysis of variance (ANOVA), followed by Tukey’s test (GraphPad Software, Inc., San Diego, CA, USA).

### 4.14. In Silico Assay

#### 4.14.1. Ligand and Target Preparations

The molecular structures of the compounds annotated from *C. grandiflora* were initially optimized at the semiempirical PM6 level using the SEQCROW module in UCSF ChimeraX v1.12. The resulting structures were parameterized with Antechamber from the AmberTools package 26.0, using the AM1-BCC charge model for the assignment of partial atomic charges and the General Amber Force Field 2 (GAFF2) for atom typing and generation of force-field parameters [68,69].

The three-dimensional crystal structure of *Leishmania infantum* trypanothione reductase (TRLi) was retrieved from the RCSB Protein Data Bank (PDB ID: 5EBK) [70]. The structure was co-crystallized with 6-(sec-butoxy)-2-((3-chlorophenyl)thio)pyrimidin-4-amine (RDS 777). Protein preparation was carried out using UCSF Chimera v1.20 and included the removal of the ligand RDS 777 and all crystallographic water molecules, followed by the addition of polar hydrogen atoms. Force-field parameters were assigned using the AMBER ff14SB force field for standard amino acid residues, whereas AM1-BCC charges were assigned to non-standard residues. Finally, the protein structure was subjected to 500 steps of steepest descent energy minimization prior to docking simulations [71].

#### 4.14.2. Molecular Docking

Molecular docking simulations were performed using AutoDock Vina-GPU 2.0. The structures of the target protein and prepared ligands were converted into the standard PDBQT format using the AutoDockTools (ADT) package 1.5.7. For the docking simulations, the receptor was kept rigid, while full conformational flexibility was allowed for the ligands. The previously assigned AMBER ff14SB and AM1-BCC partial charges, along with polar hydrogen orientations, were preserved during the receptor and ligand preparation for the docking runs.

A cubic grid box with dimensions of 25 × 25 × 25 Å along the X, Y, and Z axes was centered at the Cartesian coordinates x = 30.395, y = 49.323, z = −6.109, encompassing the active-site residues Cys52, Cys57, and Glu466′ [72]. AutoDock Vina-GPU was used to generate 100 binding poses with the following parameters: threads = 8000, search depth = 500, and energy range = 3 kcal/mol.

The most favorable ligand–receptor complexes were selected based on binding affinity, root mean square deviation (RMSD) values, visual inspection, and analysis of the residues involved in ligand–residue interactions. Molecular visualization and interaction analyses were performed using UCSF ChimeraX v1.12 and Discovery Studio 25.1 [73].

## 5. Conclusions

The present study expands the chemical and biological knowledge of *C. grandiflora* fruits and reinforces their relevance as an underexplored source of bioactive metabolites. The findings demonstrated antileishmanial activity against both promastigote and axenic amastigote forms of *L. amazonensis*, together with distinct cell death phenotypes and reduced nitric oxide production in LPS-stimulated macrophages. HPLC-ESI-IT-MS/MS analysis revealed a metabolic profile predominantly composed of putatively annotated polyprenylated benzophenones, along with a prenylated flavonoid and a pentacyclic triterpene, highlighting the chemical diversity of this species. Molecular docking was employed as an exploratory approach to prioritize putatively annotated metabolites for future investigation, without establishing TRLi inhibition or a specific mechanism of action. Future studies should focus on the isolation and structural confirmation of these metabolites, followed by biochemical assays to experimentally validate the hypotheses generated by the computational analysis.

## Figures and Tables

**Table 1:** Cytotoxic effect of the crude extract (EBCG), the dichloromethane fraction (FDCG) and the hexane fraction (FHex) against promastigote and axenic amastigote forms of *L. amazonensis* and RAW macrophages 264.7.

**Promastigote**			
**Samples**	**IC_50_ (µg mL^-1^)**	**CC_50_ (µg mL^-1^)**	**S.I.**
EBCG	>500	120.70	<0.24
FDCG	20.34	218.70	10.75
FHex	85.86	134.40	1.56
**Amastigote**			
**Samples**	**IC_50_ (µg mL^-1^)**	**CC_50_ (µg mL^-1^)**	**S.I.**
EBCG	15.92	120.70	7.58
FDCG	26.43	218.70	8.27
FHex	7.00	134.40	19.20

IC_50_: concentration required to inhibit 50% of parasite growth. CC_50_: cytotoxic concentration to 50% of RAW 264.7 macrophages. S.I.: selectivity index (CC_50_/IC_50_).

**Table 2:** Nitric oxide inhibition by EBCG, FDCG, and FHex in LPS-stimulated RAW 264.7 murine macrophages.

Samples	IC_50_ (µg mL^−1^)
EBCG	2.96 ± 0.90 ^ab^
FDCG	4.12 ± 0.67 ^ab^
FHEX	2.10 ± 0.53 ^a^
Dexamethasone	1.86 ± 0.54

The 50% inhibitory concentration of NO production, expressed as mean ± SD (*n* > 3). ^a^ Significant difference among the samples (*p* < 0.0001). ^b^ Significant difference compared to the dexamethasone control (*p* < 0.05).

## Data Availability

The original contributions presented in this study are included in the article/Supplementary Materials. Further inquiries can be directed to the corresponding author.

## Supplementary Materials

The following supporting information can be downloaded at https://www.mdpi.com/article/10.3390/molecules31162859/s1, Table S1: Annotation of the compounds of the crude extract, hexane and dichloromethane fractions of *Clusia grandiflora*. Figure S1: Mass spectrum of propolone B. Scheme S1: Proposed fragmentation of propolone B. Figure S2: Mass spectrum of prenylated kaempferol. Scheme S2: Proposed fragmentation of prenylated kaempferol. Figure S3: Mass spectrum of lupeol. Scheme S3: Proposed fragmentation of lupeol. Figure S4: Mass spectrum of propolone C. Scheme S4: Proposed fragmentation of propolone C. Figure S5: Mass spectrum of garcinielliptona I. Scheme S5: Proposed fragmentation of garcinielliptona. Figure S6: Mass spectrum of propolone D. Scheme S6: Proposed fragmentation of propolone D. Figure S7: Mass spectrum of propolone D hydroperoxide. Scheme S7: Proposed fragmentation of propolone D hydroperoxide. Figure S8: Mass spectrum of nemorosone. Scheme S8: Proposed fragmentation of nemorosone. Figure S9: Mass spectrum of garcinol. Scheme S9: Proposed fragmentation of garcinol. Figure S10: Mass spectrum of propolone A. Scheme S10: Proposed fragmentation of propolone A. Figure S11: Mass spectrum of Scrobiculatone B. Scheme S11: Proposed fragmentation of Scrobiculatone B. Figure S12: Mass spectrum of Guttiferone E. Scheme S12: Proposed fragmentation of Guttiferone E. Figure S13: Mass spectrum of Xanthocymol. Scheme S13: Proposed fragmentation of Xanthocymol. Figure S14: Concentration–response curves used for IC_50_ determination against *L. amazonensis* promastigotes for EBCG, FDCG, and FHex. IC_50_ values were estimated by nonlinear regression using GraphPad Prism 7.0. Table S2: Antileishmanial activity against *L. amazonensis* promastigotes and cytotoxicity against RAW 264.7 macrophages of the EBCG, FDCG, and FHex obtained from *C. grandiflora* fruits. Figure S15: Concentration–response curves used for IC_50_ determination against *L. amazonensis* amastigotes for EBCG, FDCG, and FHex. IC_50_ values were estimated by nonlinear regression using GraphPad Prism 7.0. Table S3: Antileishmanial activity against *L. amazonensis* promastigotes and cytotoxicity against RAW 264.7 macrophages of the EBCG, FDCG, and FHex obtained from *C. grandiflora* fruits. Figure S16: Concentration-response curves of the EBCG, FDCG, and FHex in RAW 264.7 macrophages. The curves were fitted by nonlinear regression to estimate IC_50_ values. Table S4: Binding affinity scores and residue interactions of annotated compounds of *C. grandiflora* against *L. infantum* Trypanothione reductase (TRLi; PDB ID: 5EBK). References [6,28,30,31,34,35,36] are cited in the Supplementary Materials.
