# Supplementary material for: Chemical Annotation and Biological Evaluation of Prenylated Benzophenone-Rich Fractions from Clusia grandiflora Fruits: Integrated In Vitro and In Silico Analyses of Antileishmanial Activity, Nitric Oxide Modulation, and Stage-Dependent Cell Death Phenotypes
Source: Molecules. 2026 Aug 16;31(16):2859. doi: 10.3390/molecules31162859 (PMC13515287; doi:10.3390/molecules31162859)
Supplement: Supplementary file 1 [file molecules-31-02859-s001.zip › molecules-4447542-Supplementary.pdf]

Supplementary material

# Chemical Annotation and Biological Evaluation of Prenylated Benzophenone-Rich Fractions from *Clusia grandiflora* Fruits: Integrated In Vitro and In Silico Analyses of Antileishmanial Activity, Nitric Oxide Modulation, and Stage-Dependent Cell Death Phenotypes

**Amanda de Jesus Alves Miranda [1], Caroline Martins de Jesus [2], Kassandra Gómez Valenzuela [3],**
**José Alberto Pérez Burgos [3], Samuel dos Santos Soares Buna [1], Lívia Maria Martins Carvalho [1],**
**Lucas Cardoso Marinho [4], José de Sousa Lima Neto [5], Marcelo José Dias Silva [6], Alberto Jorge Oliveira Lopes [1],**
**Lucilene Amorim Silva [2], Luisa Alondra Rascón Valenzuela [3] and Cláudia Quintino da Rocha [1,*]**

1 Natural Products Chemistry Laboratory, Department of Chemistry, Federal University of Maranhão,
São Luís 65080-805, MA, Brazil; miranda.amanda@discente.ufma.br (A.d.J.A.M.);
samuel.buna@discente.ufma.br (S.d.S.S.B.); livia.martins@alumni.ufma.br (L.M.M.C.);
lopesajo@gmail.com (A.J.O.L.)
2 Parasitology and Immunology Laboratory, Center for Biological and Health Sciences, Federal University of Maranhão, São Luís 65080-805, MA, Brazil; caroline.mj@discente.ufma.br (C.M.d.J.);
lucilene.silva@ufma.br (L.A.S.)
3 Natural Resources Research Laboratory, University of Sonora, Hermosillo 83000, Sonora, Mexico;
kassandra.gomez@unison.mx (K.G.V.); alberto.perez@unison.mx (J.A.P.B.);
luisa.rascon@unison.mx (L.A.R.V.)
4 Laboratory of the Angiosperm Systematics and Taxonomy Research Group, Department of Biology, Federal University of Maranhão, São Luís 65080-805, MA, Brazil; lcmarinho1@ufma.com
5 Laboratory for Advanced Studies in Phytomedicines, Department of Biology, Federal University of
Maranhão, São Luís 65080-805, MA, Brazil; jose.sln@ufma.br
6 Graduate Program in Pharmaceutical Care, Department of Food and Drugs, Federal University of Alfenas, Alfenas 37130-001, MG, Brazil; marcelo.dias@unifal-mg.edu.br
* Correspondence: rocha.claudia@ufma.br

Table S1: Annotation of the compounds of the crude extract, hexane and dichloromethane fractions of *Clusia grandiflora.*

| Peaks | Retention Time (Min) | Compounds | | Fragments (*m/z*) | EBCG | FHex | FDCG | References |
|---|---|---|---|---|---|---|---|---|
| | | | MS$^2$ | 553 [M+H]$^+$; **535**; 517; 399 | ✓ | - | - | |
| | | | MS$^2$ | 553 [M+H]$^+$; **535**; 399 | - | ✓ | - | |
| 1 | 11.5 | Propolone B | MS$^3$ | 553 → 535; 517; 445; 399; 381; **327**; 277 | | | | [28] |
| | | (Isomer) | MS$^2$ | 553 [M+H]$^+$; **535**; 517; 399; 327 | - | - | ✓ | |
| | | | MS$^3$ | 553 → 535; **517**; 399; 381; 327; 309; 277 | | | | |
| 2 | 14.2 | Prenylated kaempferol | MS$^2$ | 353 [M+H]$^+$;335; **285**; 267; 229 | - | ✓ | - | [30] |
| | | | MS$^3$ | 353 → 285; 267; **229**; 151 | | | | |
| | | | MS$^2$ | 427 [M+H]$^+$; **409**; 391; 191; 177 | ✓ | - | - | |
| | | | MS$^2$ | 427 [M+H]$^+$; **409**; 391; 191; 177 | - | ✓ | - | |
| 3 | 20.8 | Lupeol | MS$^3$ | 427 → 409; 391; 191; **177** | | | | [31] |
| | | | MS$^2$ | 427 [M+H]$^+$; **409**; 391; 191; 177 | - | - | ✓ | |
| | | | MS$^3$ | 427 → 409; 391; 191; **177** | | | | |
| 4 | 22.6 | Propolone C | MS$^2$ | 519 [M+H]$^+$; **451**; 397; 327 | - | ✓ | - | [28] |
| | | (Isomer) | | | | | | |
| | | Garcinielliptone I or | MS$^2$ | 519 [M+H]$^+$; **451**; 397; 359; 327 | - | ✓ | - | |
| 5 | 23.8 | hyperibone B | MS$^3$ | 519 → 451; **327** | | | | [28] |
| | | (Isomer) | MS$^2$ | 519 [M+H]$^+$; **451**; 397; 359; 327 | - | - | ✓ | |
| | | | MS$^3$ | 519 → 451; 433; **327** | | | | |
| 6 | 23.8 | Propolone D | MS$^2$ | 519 [M+H]$^+$; 501; 483; 383; 327; 309 | ✓ | - | - | [28] |
| | | (Isomer) | | | | | | |
| 7 | 24.2 | Propolone D hydroperoxide | MS$^2$ | 535 [M+H]$^+$;503; 391; **343**; 267 | - | ✓ | - | [28] |
| | | (Isomer I) | MS$^3$ | 535 → 343; 297; **267**; 189; 177 | | | | |
| 8 | 24.5 | Garcinielliptone I or | MS$^2$ | 519 [M+H]$^+$; 463; **451**; 435; 397; 327 | ✓ | - | - | [28] |
| | | hyperibone B | | | | | | |
| | | (Isomer II) | | | | | | |
| | | | MS$^2$ | 519 [M+H]$^+$; 501; 483; 383; **327** | - | ✓ | - | |

| | | | | | | | | |
|---|---|---|---|---|---|---|---|---|
| 9 | 24.7 | Propolone D | MS$^3$ | 519 → 327; 309; **255**; 249; 177 | | | | [28] |
| | | (Isomer II) | MS$^2$ | 519 [M+H]$^+$; 501; 397; 383; **327** | - | - | ✓ | |
| | | | MS$^3$ | 519 → 451; **327** | | | | |
| | | | MS$^2$ | 519 [M+H]$^+$; **451**; 397; 359; 327 | - | ✓ | - | |
| 10 | 25.5 | Garcinielliptone I or | MS$^3$ | 519 → 451; **327** | | | | [28] |
| | | hyperibone B | MS$^2$ | 519 [M+H]$^+$; 501; **451**; 397; 327 | - | - | ✓ | |
| | | (Isomer III) | MS$^3$ | 519 → 451; **327** | | | | |
| 11 | 26.8 | Garcinielliptone I or | MS$^2$ | 519 [M+H]$^+$; 501; 463; **451**; 397; 327 | - | ✓ | - | [28] |
| | | hyperibone B | MS$^3$ | 519 → 451; 433; **327**; 309 | | | | |
| | | (Isomer IV) | MS$^2$ | 519 [M+H]$^+$; 501; 463; **451**; 397; 327 | - | - | ✓ | |
| | | | MS$^3$ | 519 → 451; 433; **327** | | | | |
| | | | MS$^2$ | 503 [M+H]$^+$; 447; 435; **379**; 343; 311; 255 | ✓ | - | - | |
| | | | MS$^2$ | 503 [M+H]$^+$; 447; 435; 379; 343; 311 | - | ✓ | - | |
| 12 | 30.8 | Nemorosone | MS$^3$ | 503 → 379; **255**; 177 | | | | [28] |
| | | (Isomer) | MS$^2$ | 503 [M+H]$^+$; 447; 435; 379; 343; 311 | | | | |
| | | | MS$^3$ | 503 → **255**; 195 | | | | |
| | | | MS$^2$ | 603 [M+H]$^+$; 585; 467; **411**; 343; 287 | ✓ | - | - | |
| 13 | 31.8 | Garcinol | MS$^2$ | 603 [M+H]$^+$; 585; 467; 449; **411**; 343; 233 | - | - | ✓ | [34] |
| | | (Isomer I) | MS$^3$ | 603 → 411: **301**; 287; 195; 177 | | | | |
| | | | MS$^2$ | 503 [M+H]$^+$; 447; 435; 367; **311**; 255 | ✓ | - | - | |
| | | | MS$^2$ | 503 [M+H]$^+$; 447; 435; 367; **311**; 255 | - | ✓ | - | |
| 14 | 40.1 | Propolone A | MS$^3$ | 503 → 311; **255** | | | | [28] |
| | | (Isomer I) | MS$^2$ | 503 [M+H]$^+$; 485; 447; 435; 367; **311**; 255 | - | - | ✓ | |
| | | | MS$^3$ | 503 → 311; **255** | | | | |
| | | | MS$^2$ | 501 [M+H]$^+$; 445; 433; **309**; 255 | ✓ | | | |
| | | | MS$^2$ | 501 [M+H]$^+$; 445; 433; 341; **309**; 217 | | | | |
| 15 | 40.3 | Scrobiculatone B | MS$^3$ | 501→309; 291; **231** | | | | [6] |
| | | (Isomer I) | MS$^2$ | 501 [M+H]$^+$; 445; 433; 341; **309** | | ✓ | | |
| | | | MS$^3$ | 501 → 309: 291; 255; **231** | | | | |
| | | | MS$^2$ | 503 [M+H]$^+$; 435; 379; **311** | ✓ | - | - | |
| 16 | 41.1 | Propolone A | MS$^2$ | 503 [M+H]$^+$; 447; **435**; 379; 367; 343; 311 | - | ✓ | - | [28] |

| | | | | | | | | |
|---|---|---|---|---|---|---|---|---|
| | | (Isomer II) | MS$^3$ | 501 → 435: **311** | | | | |
| 17 | 41.3 | Scrobiculatone B | MS$^2$ | 501 [M+H]$^{+}$; 445; 433; 377; 365; 341; **309** | ✓ | - | - | [6] |
| | | (Isomer II) | MS$^3$ | 501 → 309; **231** | | | | |
| 18 | 41.4 | Propolone A | MS$^2$ | 503 [M+H]$^{+}$; 447; 435; 379); **311**; 255 | - | - | ✓ | [28] |
| | | (Isomer III) | MS$^3$ | 503→311; **255** ; 233 | | | | |
| | | | MS$^2$ | 503 [M+H]$^{+}$; 435; 379; **311**; 255 | ✓ | - | - | |
| 19 | 41.8 | Propolone A | MS$^2$ | 503 [M+H]$^{+}$; **435**; 367; 343; 311 | - | ✓ | - | [28] |
| | | (Isomer IV) | MS$^3$ | 503→435; **311** | | | | |
| | | | MS$^2$ | 501 [M+H]$^{+\cdot}$;445; 433; 377; **365**; 341; 309 | - | ✓ | - | |
| 20 | 42.2 | Scrobiculatone B | MS$^3$ | 501 → 309; **231** | | | | [6] |
| | | (Isomer III) | MS$^2$ | 501 [M+H]$^{+\cdot}$; 445; 433; 379; 309 | - | - | ✓ | |
| 21 | 43.2 | Guttiferone E | MS$^2$ | 603 [M+H]$^{+\cdot}$; 585; 549; 467; **411**; 343; 287 | ✓ | - | - | [35,36] |
| 22 | 43.4 | Xanthocymol | MS$^2$ | 603 [M+H]$^{+\cdot}$; 585; 549; 467; **411**; 343 | ✓ | - | - | [35,36] |
| | | | MS$^2$ | 603 [M+H]$^{+}$; 585; 535; 467; 449; **411**; 343; 287; 233 | - | ✓ | - | |
| 23 | 44.9 | Garcinol (Isomer II) | MS$^3$ | 603 → 411; **301**; 287; 177 | | | | [34] |
| | | | MS$^2$ | 503 [M+H]$^{+}$; 485; 447; 435; 379; 367; 343; **311**; 275; 255 | - | ✓ | - | |
| 24 | 49.5 | Propolone A | MS$^3$ | 503 → 311; **255**; 233 | | | | [28] |
| | | (Isomer V) | MS$^2$ | 503 [M+H]$^{+}$; 485; 447; 435; 379; 367; 343; **311;** 275; 255 | - | - | ✓ | |
| | | | MS$^3$ | 503 → 311; **255**; 231 | | | | |
| | | | MS$^2$ | 503 [M+H]$^{+}$; 447; 435; 379; 367; 343; **311** | - | ✓ | - | |
| 25 | 50.7 | Propolone A | MS$^3$ | 503 → 311; **255**; 233 | | | | [28] |
| | | (Isomer VI) | MS$^2$ | 503 [M+H]$^{+}$; 447); 435; 379; 367; 343; **311** | - | - | ✓ | |
| | | | MS$^3$ | 503 → 311; **255**; 233 | | | | |

MS2 and MS3 spectra of compounds annotated in EBCG, FDCG and FHex from *Clusia grandiflora.*

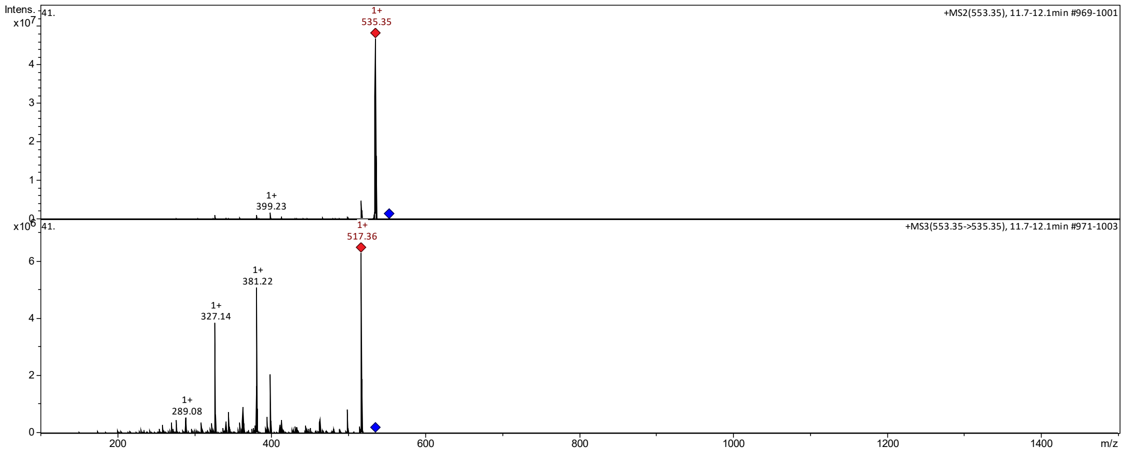


**Figure S1.** Mass spectrum of propolone B

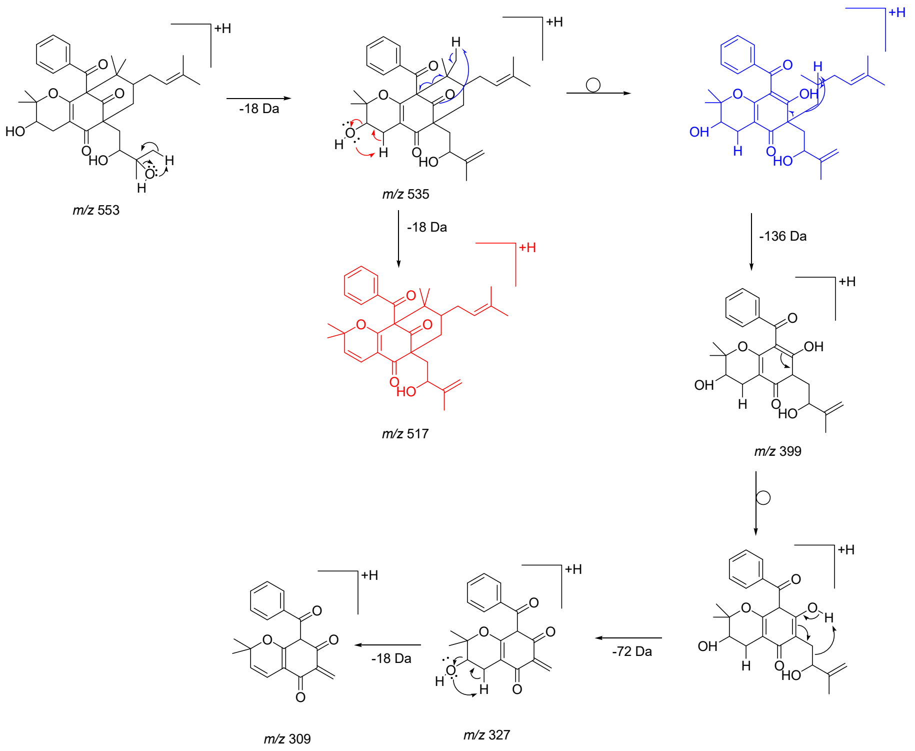


**Scheme S1.** Proposed fragmentation of propolone B

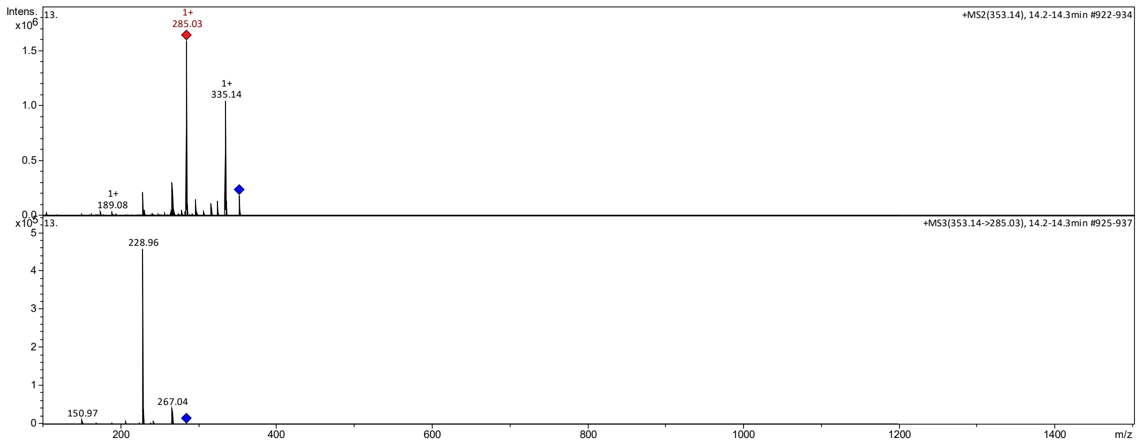


**Figure S2.** Mass spectrum of prenylated kaempferol

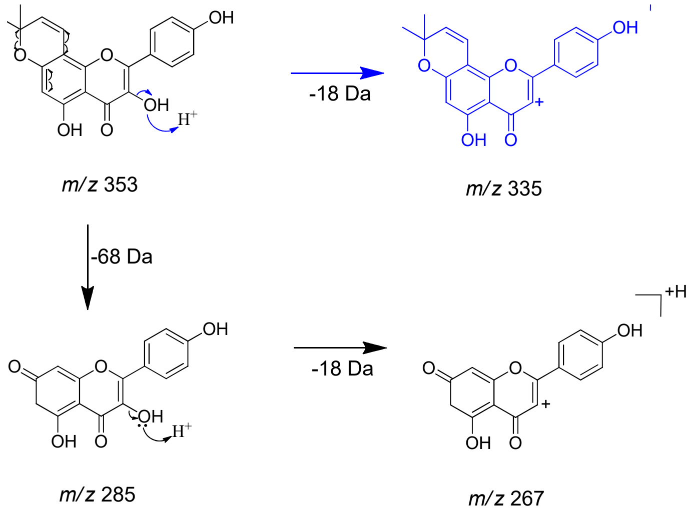


**Scheme S2.** Proposed fragmentation of prenylated kaempferol

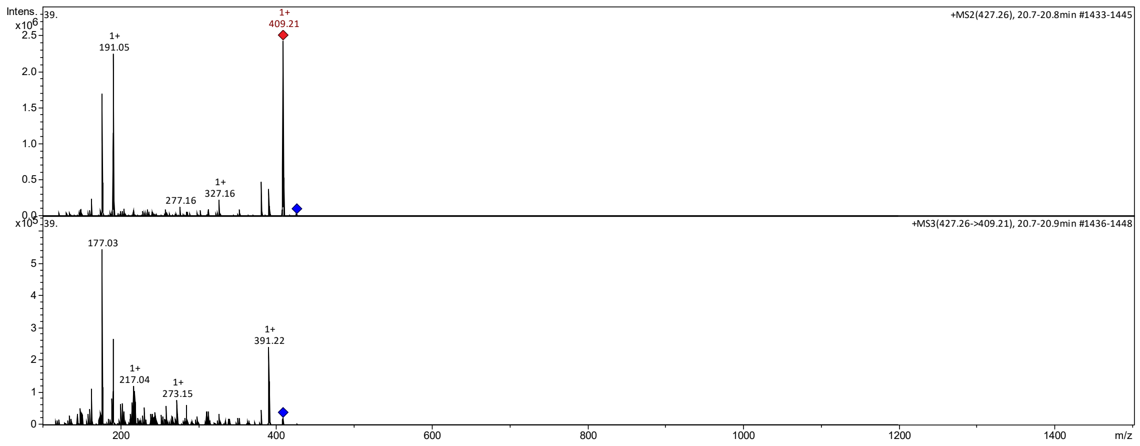


**Figure S3.** Mass spectrum of lupeol

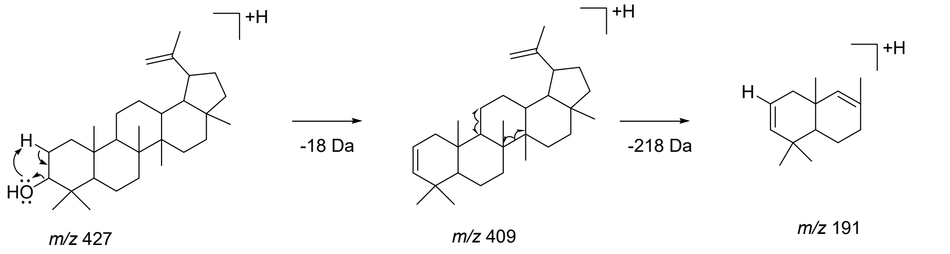


**Scheme 3.** Proposed fragmentation of lupeol

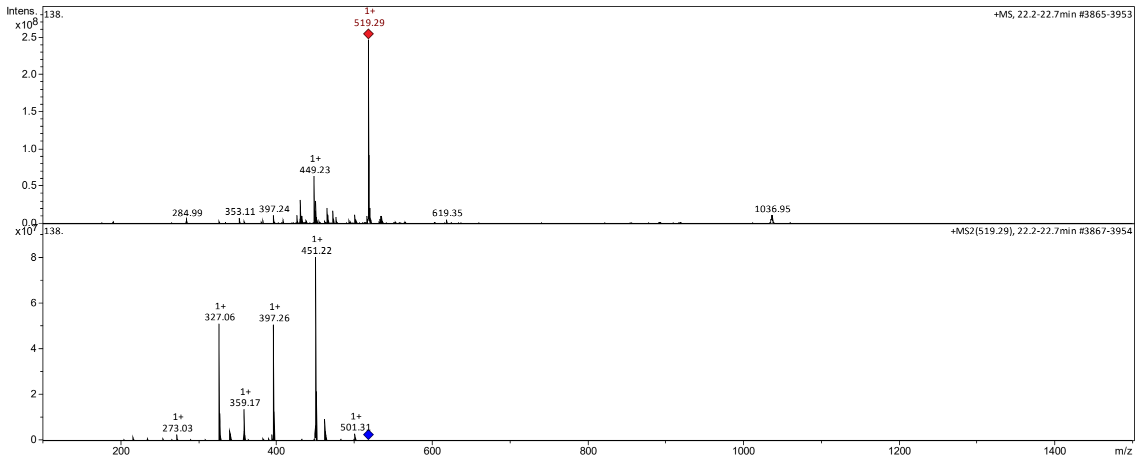


**Figure S4.** Mass spectrum of propolone C

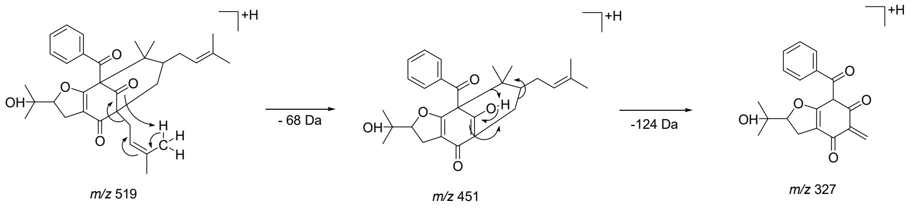

**Scheme S4.** Proposed fragmentation of propolone C

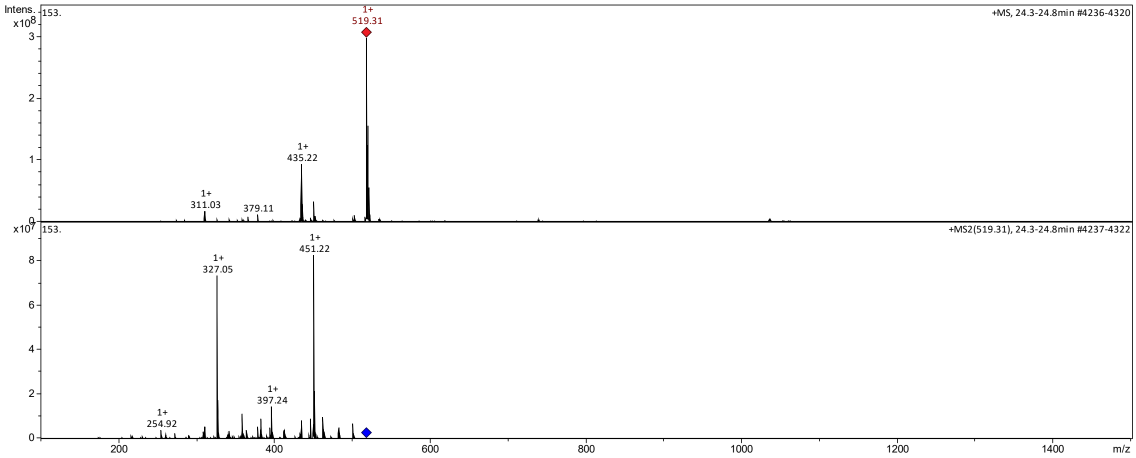


**Figure S5.** Mass spectrum of garcinielliptona I

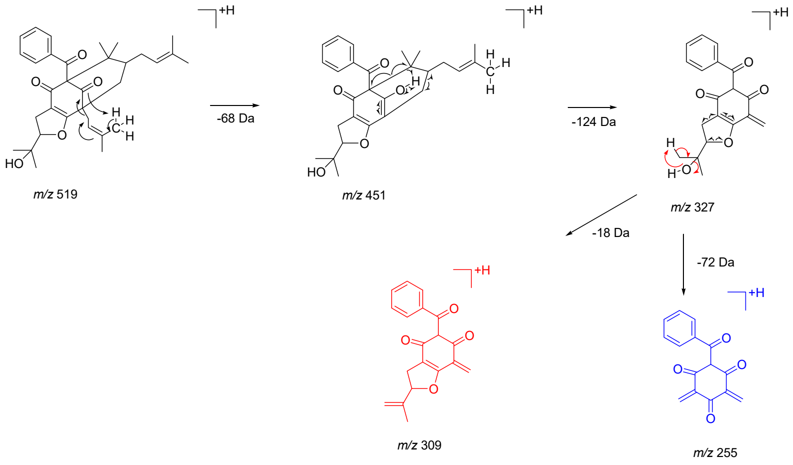


**Scheme S5.** Proposed fragmentation of garcinielliptona

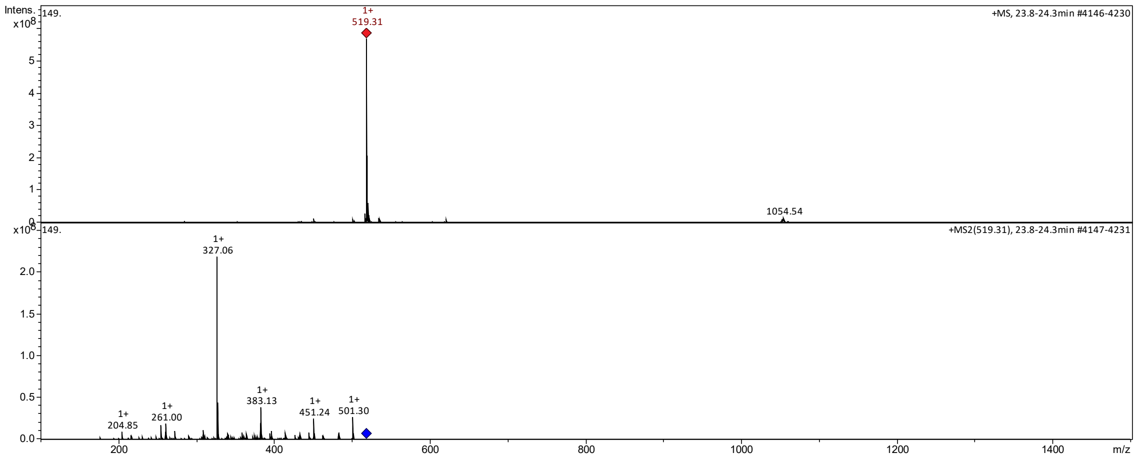

**Figure S6.** Mass spectrum of propolone D

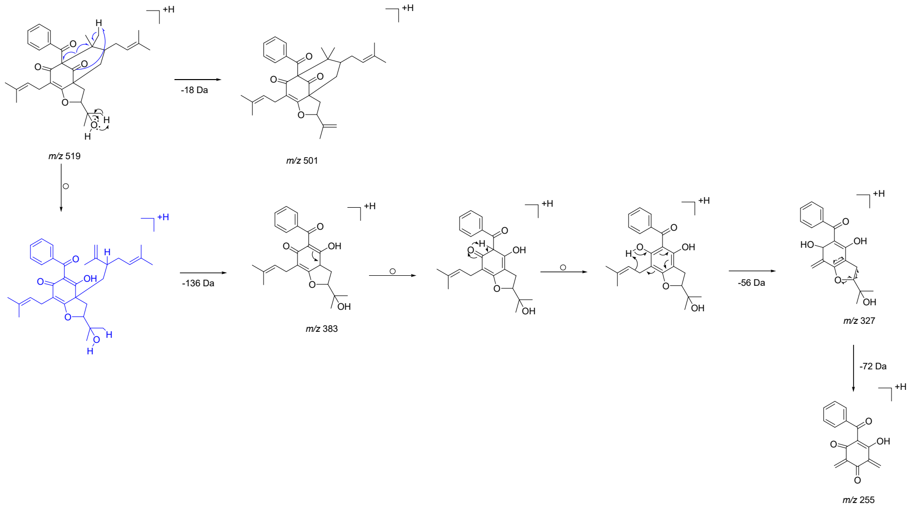


**Scheme S6.** Proposed fragmentation of propolone D

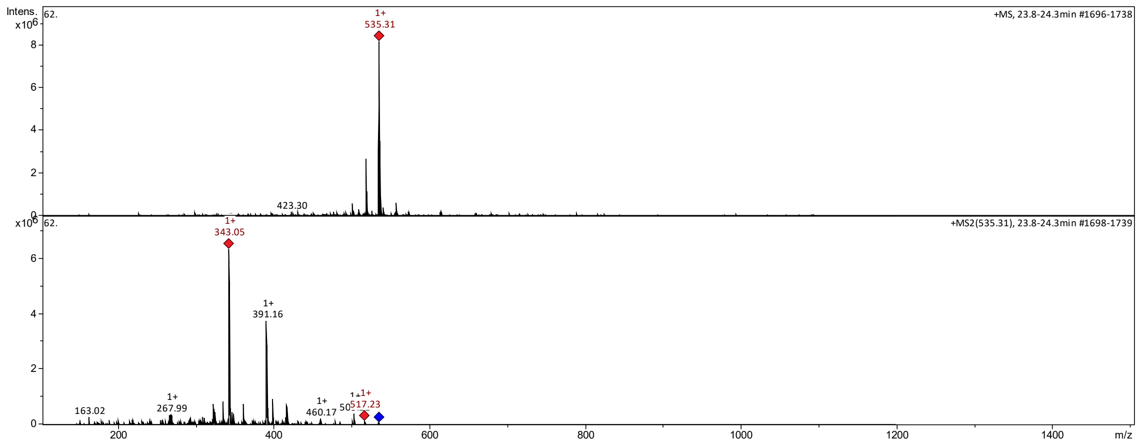


**Figure S7.** Mass spectrum of propolone D hydroperoxide

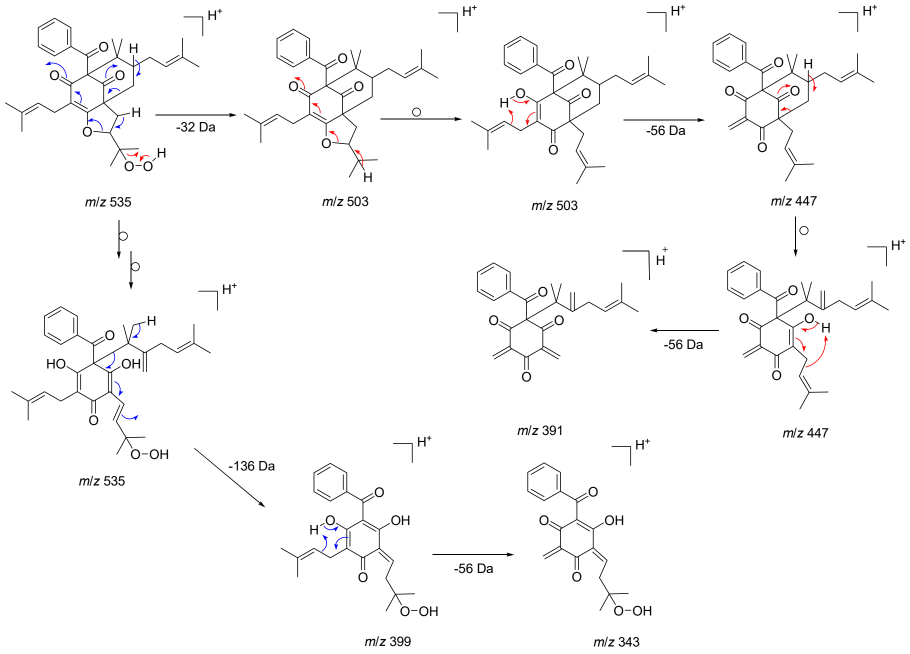


**Scheme S7.** Proposed fragmentation of propolone D hydroperoxide

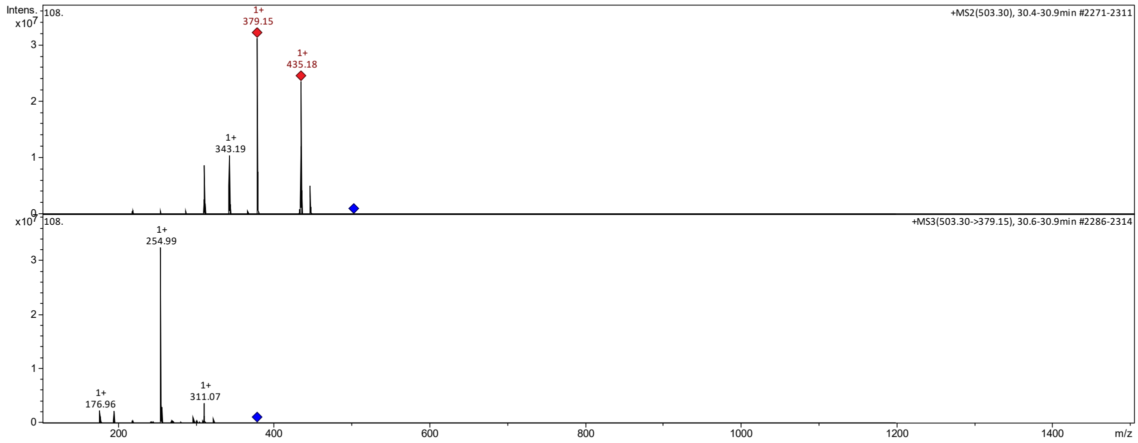


**Figure S8.** Mass spectrum of nemorosone

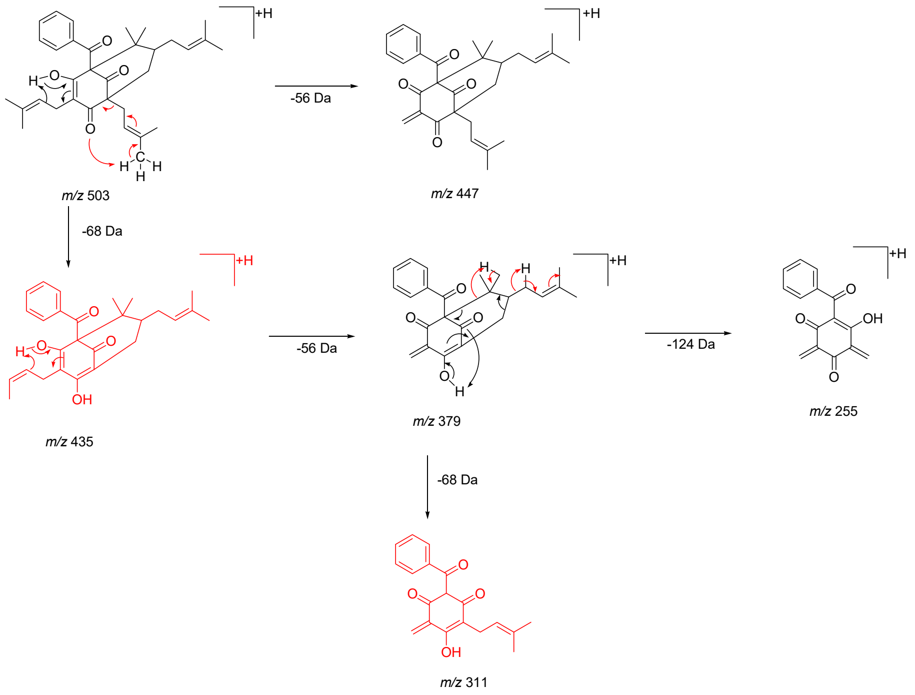


**Scheme S8.** Proposed fragmentation of nemorosone

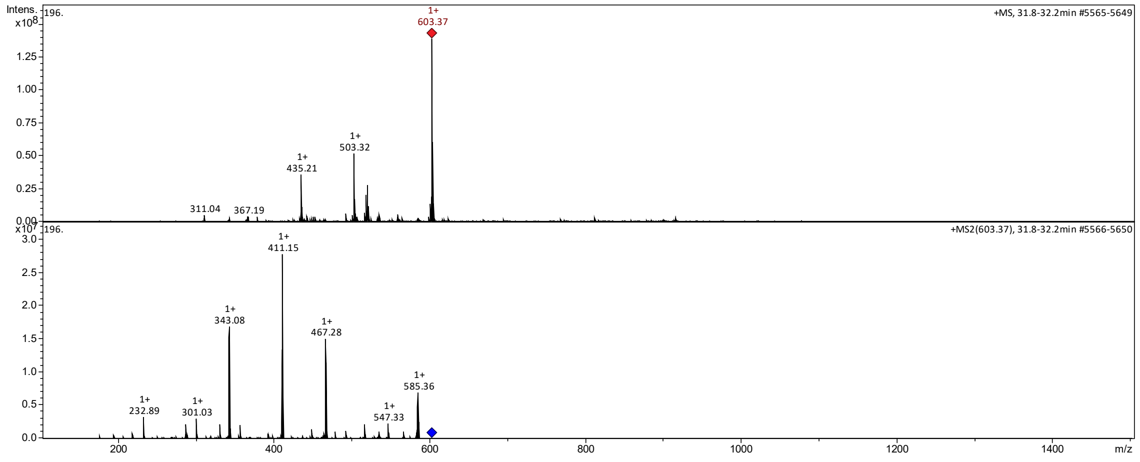


**Figure S9.** Mass spectrum of garcinol

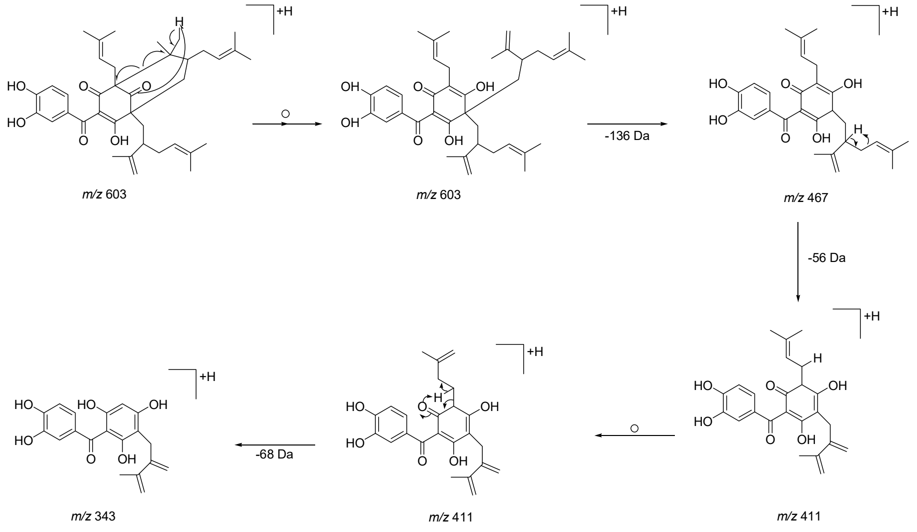


**Scheme S9.** Proposed fragmentation of garcinol

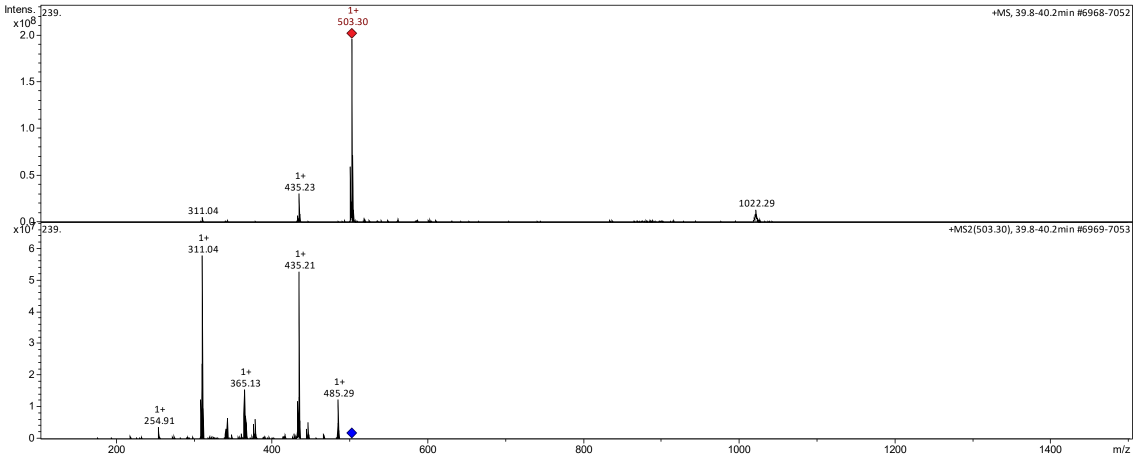


**Figure S10.** Mass spectrum of propolone A

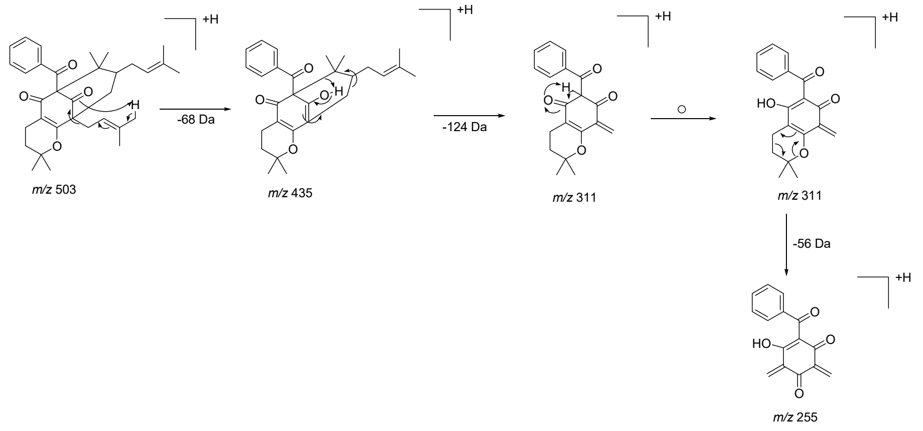


**Scheme S10.** Proposed fragmentation of propolone A

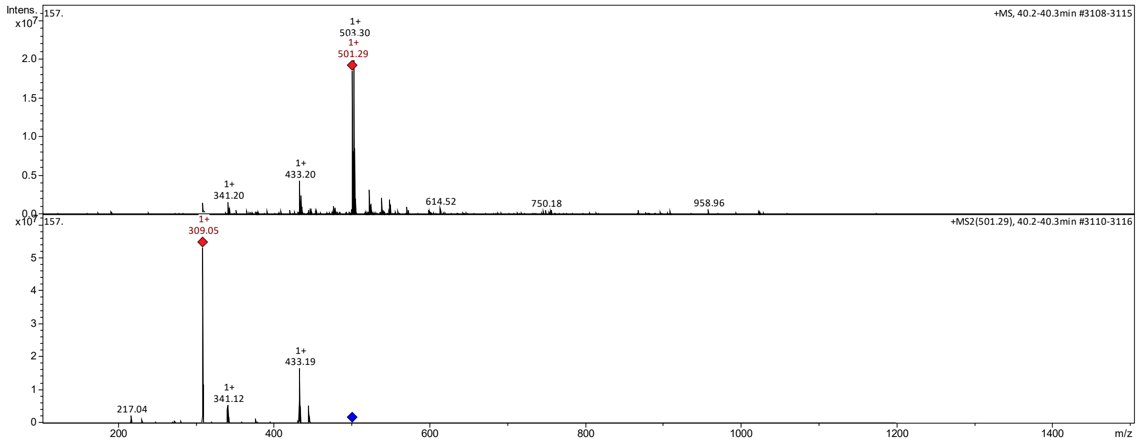


**Figure S11.** Mass spectrum of Scrobiculatone B

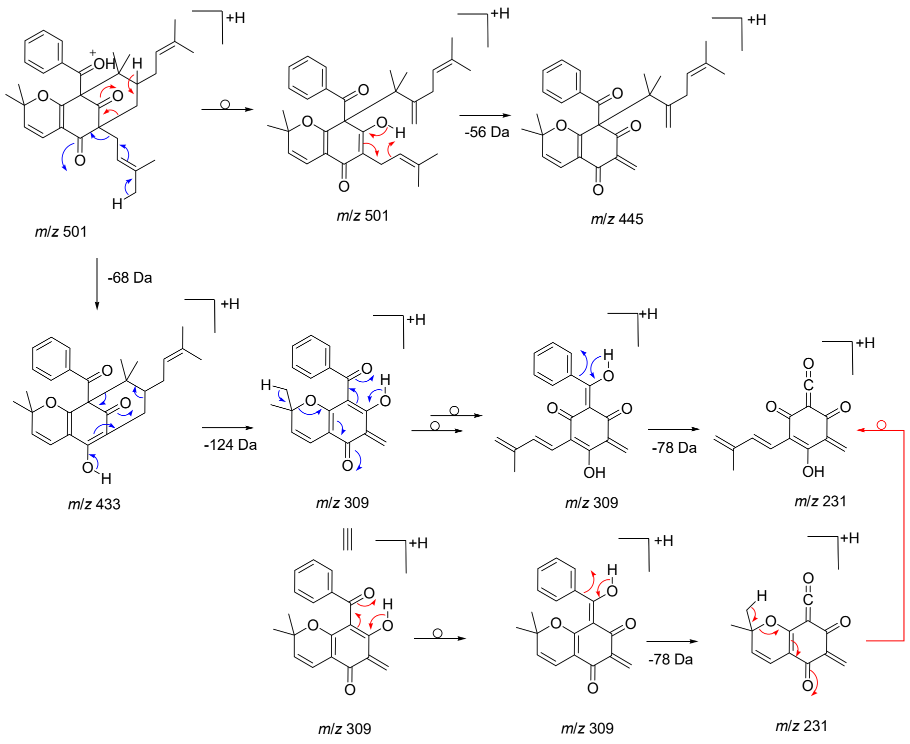


**Scheme S11.** Proposed fragmentation of Scrobiculatone B

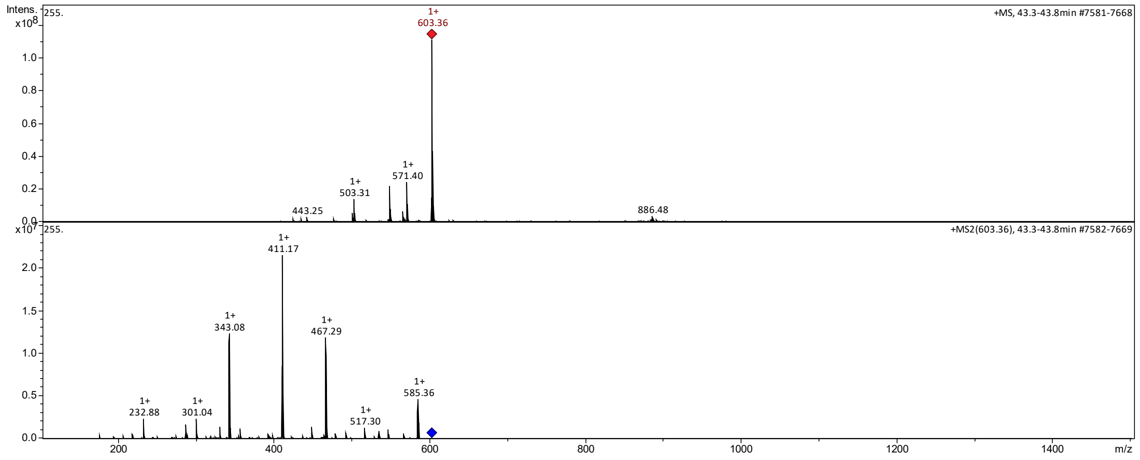


**Figure S12.** Mass spectrum of Guttiferone E

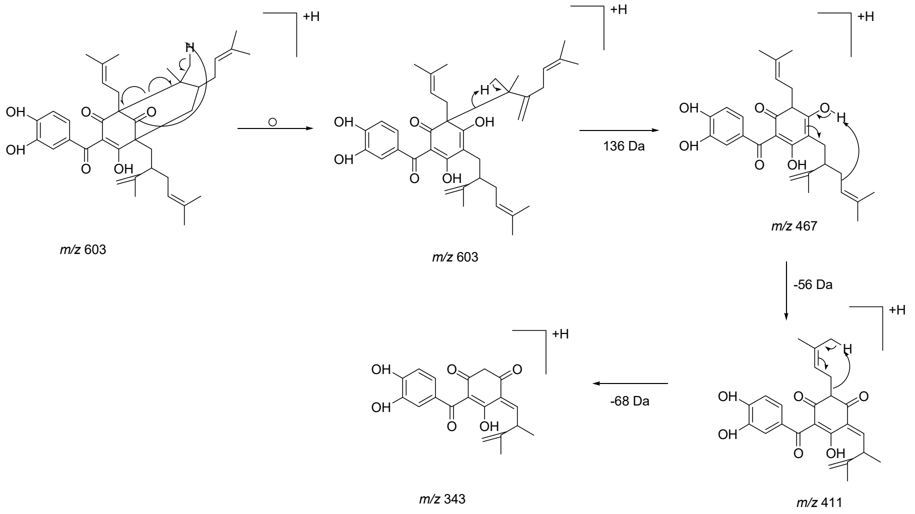


**Scheme S12.** Proposed fragmentation of Guttiferone E

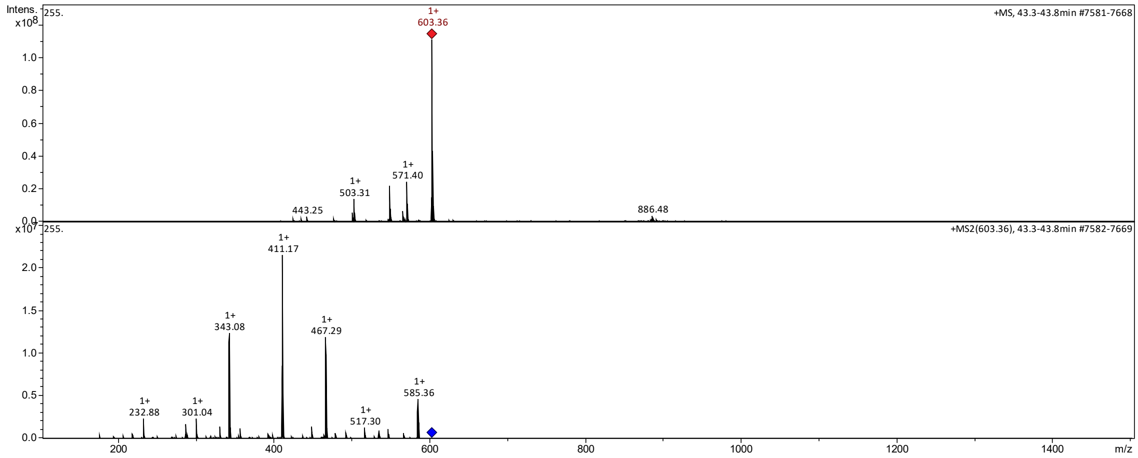


**Figure S13.** Mass spectrum of Xanthocymol

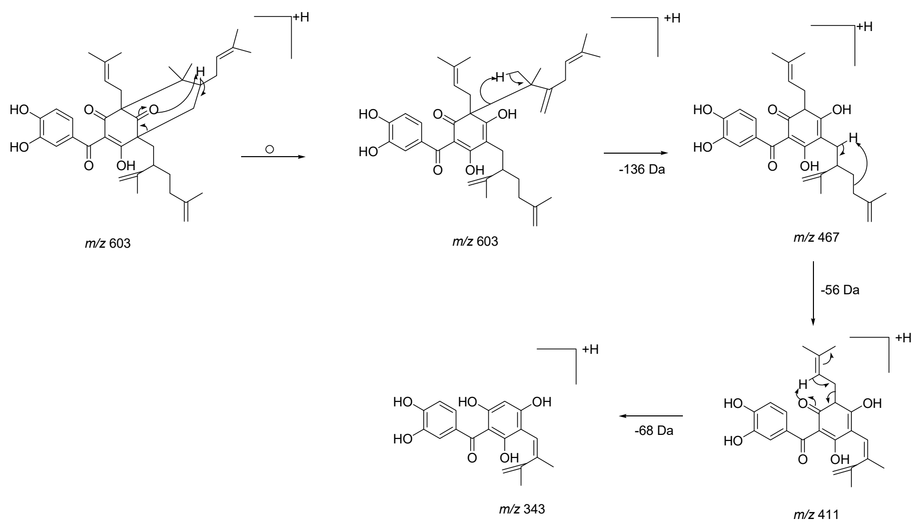


**Scheme S13.** Proposed fragmentation of Xanthocymol

**Figure S14**. Concentration–response curves used for $IC_{50}$ determination against *L. amazonensis* promastigotes for EBCG), FDCG, and FHex. $IC_{50}$ values were estimated by nonlinear regression using GraphPad Prism 7.0.

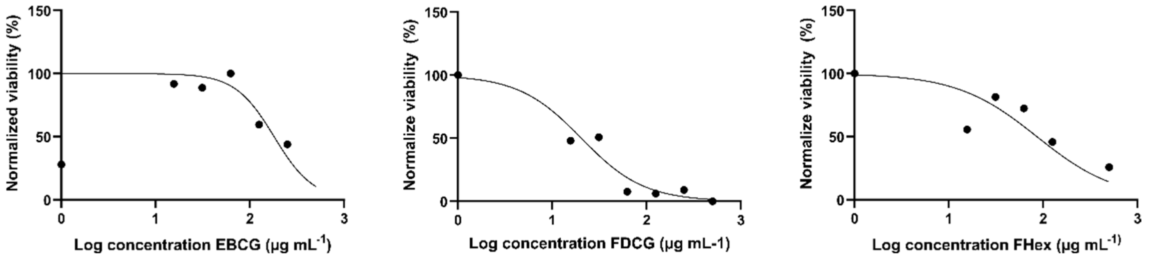


**Table S2.** Antileishmanial activity against *L. amazonensis* promastigotes and cytotoxicity against RAW 264.7 macrophages of the EBCG, FDCG, and FHex obtained from *C. grandiflora* fruits.

| Samples | $IC_{50}$ (μg mL$^{-1}$) | 95% $CI_{50}$ | $CC_{50}$ (μg mL$^{-1}$) | 95% CI (μg mL$^{-1}$) |
|---|---|---|---|---|
| EBCG | >500 | Undefined | 120.70 | 85.97 – 165.80 |
| FDCG | 20.34 | 14.23 – 26.48 | 218.70 | 180.25 – 265.45 |
| FHex | 85.86 | 51.74 – 128.40 | 134.40 | 92.02 – 195.08 |

**Figure S15.** Concentration–response curves used for $IC_{50}$ determination against *L. amazonensis* amastigotes for EBCG, FDCG, and FHex. $IC_{50}$ values were estimated by nonlinear regression using GraphPad Prism 7.0.

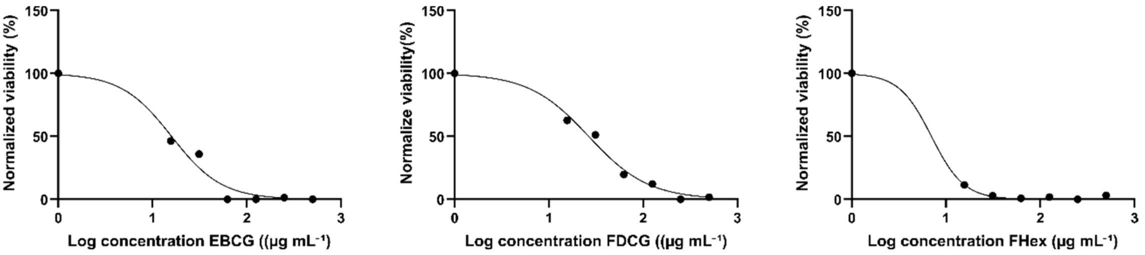


**Table S3.** Antileishmanial activity against *L. amazonensis* promastigotes and cytotoxicity against RAW 264.7 macrophages of the EBCG, FDCG, and FHex obtained from *C. grandiflora* fruits.

| Samples | $IC_{50}$ (μg mL$^{-1}$) | 95% $CI_{50}$ | $CC_{50}$ (μg mL$^{-1}$) | 95% CI (μg mL$^{-1}$) |
|---|---|---|---|---|
| EBCG | 15.92 | 13.39 –18.30 | 120.70 | 85.97 –165.80 |
| FDCG | 26.43 | 23.82 – 29.14 | 218.70 | 180.25 – 265.45 |
| FHex | 7.00 | 5.01 –7.20 | 134.40 | 92.02 – 195.08 |

**Figure S16.** Concentration-response curves of the EBCG, FDCG, and FHex in RAW 264.7 macrophages. The curves were fitted by nonlinear regression to estimate $CC_{50}$ values.

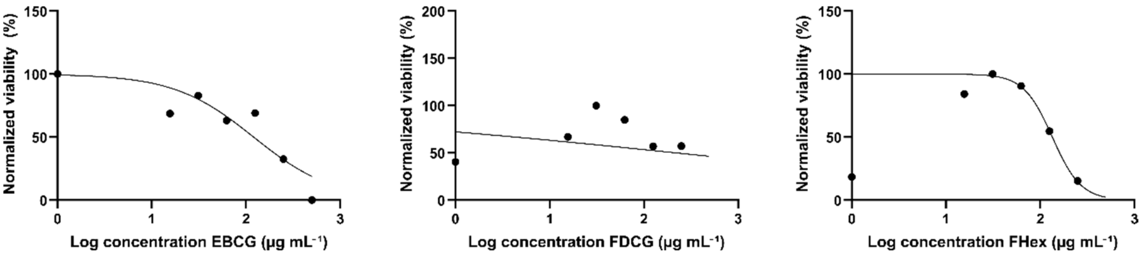


**Table S4**. Binding affinity scores and residue interactions of annotated compounds of *C. grandiflora* against *L. infantum* Trypanothione reductase (TRLi; PDB ID: 5EBK).

| Ligand | Binding affinity (kcal/mol) | RMSD (Å) | Hydrogen bond interaction | Residues interactions |
|---|---|---|---|---|
| RDS 777 | -6.6 | 1.10 | Thr463′, Glu466′ | Cys52, Cys57, Ile339, His461′ |
| Propolone B | -6.4 | 1.02 | Thr463′ | Ile106, Leu399′, Pro462′, Glu467′ |
| Prenylated Kaempferol | -6.5 | 0.55 | Ser14 | Ile339, Ile106, Val58, Leu399′, His461′ |
| Lupeol | -7.2 | 1.49 | Ser14, Tyr110 | Ile339, Phe396′, Leu399′ |
| Propolone C | -7.4 | 1.76 | Ser14, Gly49 | Ile106, Ile339, Leu399′, Phe396′, His461′ |

| | | | | |
|---|---|---|---|---|
| Garcinielliptone I | -8.0 | 0.62 | His461′ | Ile339, Ile106, Val58, Val53, Leu399′ |
| Propolone D | -7.2 | 1.14 | His461′ | Ile339, Val53, Ile106, Pro462′, Leu399′, Pro398′, Phe396′ |
| Propolone D hydroperoxide | -7.5 | 0.63 | His461′ | Ile339, Leu17, Tyr110, Ile106, Leu399′, Phe396′, Glu467′ |
| Nemerosone | -7.2 | 1.83 | - | Ile106, Tyr110, Val53, Leu399′ |
| Garcinol | -6.7 | 1.15 | His461′ | Ile106, Ile339, Val102, Val53, Tyr110, Leu399′, Phe396′, Pro398′, Pro462′ |
| Guttiferone E | -8.0 | 1.15 | Tyr110 | Ile106, Ile339, Val102, Val53, Pro398′, Phe396′, His461′ |
| Xanthocymol | -7.5 | 1.62 | - | Cys52, Cys57, Val53, Ile339, Tyr110, Ile106, Leu399′ |
| Propolone A | -7.1 | 1.76 | His461′ | Ile339, Val53, Tyr110, Leu17, Glu466′ |
| Scrobiculatone B | -7.9 | 1.23 | - | Leu399′, Pro462′, Pro398′, Phe396′, Glu466′ |
